# Fear of Missing Out and Problematic Social Media Use Among Chinese University Students: Latent Profiles and Two-Wave Network Comparisons

**DOI:** 10.3390/bs16050678

**Published:** 2026-04-29

**Authors:** Yang Wang, Lei Zhang, Jon D. Elhai, Christian Montag, Haibo Yang

**Affiliations:** 1 Zhejiang Provincial Clinical Research Center for Mental Health, The Affiliated Kangning Hospital of Wenzhou Medical University, Wenzhou 325000, China; 2 Academy of Psychology and Behavior, Faculty of Psychology, Tianjin Normal University, No. 393 Binshuixi Road, Xiqing District, Tianjin 300387, China; 3 Department of Psychology, University of Toledo, Toledo, OH 43606, USA; 4 Department of Neurosciences and Psychiatry, University of Toledo, Toledo, OH 43606, USA; 5 Centre for Cognitive and Brain Sciences, Institute of Collaborative Innovation, University of Macau, Macau SAR, China; 6 Department of Computer and Information Science, Faculty of Science and Technology, University of Macau, Macau SAR, China; 7 Department of Psychology, Faculty of Social Sciences, University of Macau, Macau SAR, China; 8 Tianjin Social Science Laboratory of Students’ Mental Development and Learning, Tianjin 300387, China

**Keywords:** fear of missing out, problematic social media use, latent profile analysis, network analysis, university students

## Abstract

Fear of missing out (FoMO) is a cognitive-affective factor that has been consistently linked to problematic social media use (PSMU), but less is known about whether this association differs across severity-based subgroups or changes over time at the node level. This study examined the cross-sectional and two-wave associations between FoMO and PSMU in Chinese university students. Two-wave data were collected one year apart from 853 participants at Time 1 and 817 participants at Time 2. Partial correlation and regression analyses showed that FoMO was positively associated with PSMU. Latent profile analysis identified broad higher- and lower-level subgroups for both FoMO and PSMU. Node-level network analyses further indicated that FoMO and PSMU nodes were positively interconnected. Most subgroup and two-wave network comparisons suggested that overall network structure was relatively stable. The clearest temporal difference emerged in the global strength of the PSMU network. When differences were observed, they were more evident in the relative prominence of specific nodes, including several bridging nodes, than in broader network organization. Overall, the findings suggest that the FoMO-PSMU association is robust, whereas subgroup- and time-related variation appears limited and is better understood as node-level variation within a broader pattern of structural stability.

## 1. Introduction

The widespread use of social media has made online interaction a routine part of daily life for many university students. At the same time, increasing evidence suggests that problematic social media use (PSMU) should be distinguished from frequent but nonproblematic use. PSMU is generally characterized by reduced control, persistent preoccupation, and continued engagement despite negative consequences (Brand et al., 2019, 2022; Shannon et al., 2022). This distinction is important because PSMU, rather than simple use frequency, has been more consistently associated with poorer psychological adjustment and functional difficulties in adolescents and young adults (Shannon et al., 2022; Wu et al., 2024). In this context, fear of missing out (FoMO), defined as a pervasive apprehension that others may be having rewarding experiences from which one is absent, has received increasing attention as a cognitive-affective factor related to problematic digital-media use (Przybylski et al., 2013). Recent studies have continued to support a positive association between FoMO and PSMU (Hou et al., 2026; Servidio et al., 2025).

The Interaction of Person-Affect-Cognition-Execution (I-PACE) model provides a useful framework for understanding this association. According to this model, addictive online behaviors may emerge through interactions among predisposing characteristics, affective and cognitive responses to internal and external triggers, and executive-control processes (Brand et al., 2016, 2019). Within this framework, FoMO can be viewed as a cognitive-affective factor that may be relevant to the development and maintenance of problematic social media use, although the present study does not test the full process assumptions of the I-PACE model (Brand et al., 2019; Przybylski et al., 2013). Although previous studies have generally reported positive associations between FoMO and PSMU, much of this work has relied on cross-sectional designs. As a result, less is known about whether the node-level organization of the FoMO-PSMU association remains stable over time or changes across measurement waves within the same population (Hou et al., 2026; Wu et al., 2024).

Addressing these questions may require methodological approaches that are sensitive to both heterogeneity and node-level organization. Many previous studies have relied on total scores and variable-centered analyses. Although these approaches are informative, they may overlook meaningful differences in how FoMO and PSMU cluster within individuals and how specific nodes are interconnected. Person-centered approaches such as latent profile analysis (LPA) can help identify subgroups with relatively higher and lower severity levels and may therefore provide a more refined understanding of risk patterns. Recent studies suggest that PSMU can vary across latent profiles and that FoMO may be associated with higher-risk profiles (Halaris et al., 2025; Servidio et al., 2025; J. Li et al., 2024). However, existing profile-based findings in this area have more often reflected severity gradients than clearly distinct qualitative subtypes, which calls for cautious interpretation of subgroup labels. In parallel, network analysis makes it possible to examine how individual nodes are related to one another and to identify nodes that may occupy relatively central or bridging positions within the broader network (Borsboom, 2017; Robinaugh et al., 2020). Network comparison procedures further allow researchers to examine whether networks differ across groups or across time in overall structure or global connectivity (Conkey-Morrison et al., 2025; van Borkulo et al., 2023).

Taken together, the combined use of LPA and network analysis may help clarify two issues that remain insufficiently understood. First, it remains unclear whether the FoMO-PSMU association differs meaningfully across broadly defined higher- and lower-severity subgroups. Second, it remains unclear whether two-wave differences are better characterized as broad structural reorganization or as more limited shifts in the relative prominence of specific nodes. In the present study, LPA was used primarily to distinguish broad severity-based subgroups rather than to assume qualitatively distinct latent types, whereas network analysis was used to examine whether node interconnections appeared broadly similar or descriptively different across these subgroups and across two measurement waves. Accordingly, we examined the relationship between FoMO and PSMU in Chinese university students using complementary person-centered and network-based approaches. First, we tested whether FoMO was positively associated with PSMU. Second, we examined whether severity-based FoMO and PSMU subgroups differed in the relative prominence of specific nodes or in overall network characteristics. Third, we examined whether the relative prominence of specific FoMO and PSMU nodes, as well as broader network characteristics, differed across two waves of data collected one year apart. Based on the I-PACE model and prior evidence, we proposed the following research questions:

RQ1: What is the association between FoMO and PSMU?

RQ2: Do severity-based FoMO and PSMU subgroups differ in the relative prominence of specific nodes or in overall network characteristics?

RQ3: Do FoMO and PSMU networks show evidence of change in node prominence or overall network organization across two waves?

## 2. Materials and Methods

### 2.1. Participants and Procedure

Data were collected through an online questionnaire hosted on www.wjx.cn (accessed from 8 to 14 December 2022 at T1 and from 13 to 23 December 2023 at T2) and distributed through social networking sites at universities in Tianjin and Shandong, China, across two time periods. On the first page of the questionnaire, participants provided informed consent by clicking a button online. Participants reported demographic information including age and gender, and completed the Chinese versions of the Bergen Social Media Addiction Scale (BSMAS; Andreassen et al., 2012) and the Fear of Missing Out (FoMO) scale (Przybylski et al., 2013). Both instruments have shown acceptable psychometric properties in Chinese samples (Luo et al., 2021; Wang et al., 2022). 

At Time 1 (T1, December 2022), 912 college students from four universities participated based on convenience sampling. After excluding incomplete or invalid responses, the final T1 sample included 853 participants (valid response rate = 93.53%), including 350 males and 503 females aged 18–36 years (*M_age_* = 20.72, *SD* = 2.40). At Time 2 (T2, December 2023), 853 students who participated at T1 completed the follow-up survey. After excluding incomplete or invalid responses, the final T2 sample included 817 participants (valid response rate = 95.78%), including 319 males and 498 females aged 18–23 years (*M_age_* = 20.15, *SD* = 1.39). To enable longitudinal data linkage while protecting participant confidentiality, T1 and T2 responses were matched using student identification numbers that were used only for data-matching purposes and were removed after matching.

The study procedures were conducted in accordance with the Declaration of Helsinki and were approved by the Ethics Committee of Tianjin Normal University (No. 2022031501). All participants were informed about the study and provided informed consent.

### 2.2. Measures

#### 2.2.1. Problematic Social Media Use

The Chinese version of the Bergen Social Media Addiction Scale (BSMAS; Luo et al., 2021; Andreassen et al., 2012) was used to assess the severity of PSMU. The scale consists of six items rated on a 5-point Likert scale (1 = very rarely, 5 = very often), with higher scores indicating higher levels of PSMU. The original scale demonstrated good internal consistency (*α* = 0.91; Luo et al., 2021). In the present sample, the BSMAS demonstrated an unidimensional structural validity, with exploratory factor analysis supporting a single-factor solution (*KMO* = 0.877; variance explained = *59.57%*; loadings = 0.725–0.797) and confirmatory factor analysis showing fit (*RMSEA* = 0.013, *CMIN/DF* = 1.835, *NFI* = 0.995, *RFI* = 0.987, *IFI* = 0.998, *CFI* = 0.998). Cronbach’s *α* for the BSMAS was 0.86 at T1 and 0.81 at T2.

#### 2.2.2. Fear of Missing Out

FoMO was assessed using the Chinese version of the FoMO scale (Wang et al., 2022), a 10-item self-report measure rated on a 5-point Likert scale (1 = not at all true of me, 5 = extremely true of me). Higher scores indicate higher levels of FoMO. The original scale demonstrated adequate internal consistency (*α* = 0.84; Wang et al., 2022). In the present sample, the FoMO demonstrated an unidimensional structural validity, with exploratory factor analysis supporting a single-factor solution (*KMO* = 0.885; variance explained = 45.84%; loadings = 0.540–0.768) and confirmatory factor analysis showing fit (*RMSEA* = 0.047, *CMIN/DF* = 2.888, *NFI* = 0.989, *RFI* = 0.962, *IFI* = 0.993, *CFI* = 0.993). Cronbach’s *α* was 0.87 at both T1 and T2.

### 2.3. Statistical Analysis

No missing item-level data occurred because the online questionnaire required a response to each item before submission. Descriptive statistics and reliability analyses were conducted using SPSS 26.0. Latent profile analysis (LPA) was conducted using Mplus 8.3. Network estimation and network comparison analyses were conducted in R 4.3.1.

First, partial correlation and hierarchical regression analyses were conducted to examine the association between FoMO and PSMU while controlling for age and gender. The regression analysis was used as a complementary construct-level analysis based on summed scale scores, whereas the network analyses were conducted at the item level using methods appropriate for ordinal questionnaire data.

Second, LPA was conducted separately for FoMO and PSMU to identify broad severity-based subgroups with relatively higher and lower levels of each construct. Model selection was based on the Akaike Information Criterion, Bayesian Information Criterion, sample-size-adjusted Bayesian Information Criterion, entropy, the Lo-Mendell-Rubin test, the Bootstrap Likelihood Ratio Test, subgroup size, and substantive interpretability. In addition to statistical fit indices, the retained solution was required to show adequate classification quality, no trivially small classes, and a practically interpretable distinction that could be meaningfully used in subsequent subgroup network analyses.

Third, network analyses were conducted at the item level because the network approach focuses on associations among individual items represented as nodes, rather than only on total scores. Networks were estimated as regularized partial correlation networks in R using the qgraph and bootnet packages. Given that the questionnaire items were ordinal, correlations were estimated using methods appropriate for ordered categorical data. The final networks were estimated using EBICglasso, in which edges represent regularized partial correlations after controlling for all other nodes in the model. Bridge centrality indices were estimated for the combined FoMO-PSMU network to identify nodes that were relatively important in linking the FoMO and PSMU communities. The subgroup analyses were used to examine whether severity-based groups differed in the relative prominence of specific nodes or in broader network characteristics. Two-wave network comparisons were then conducted to examine whether FoMO and PSMU networks differed across measurement waves based on participants who completed both waves of the study.

To improve transparency, supplementary analyses of edge-weight accuracy and centrality stability were conducted using bootstrap procedures, and the detailed results are reported in the Supplementary Materials. Given concerns about the interpretability and stability of some centrality indices in psychological networks, greater emphasis was placed on strength and bridge strength, whereas closeness and betweenness were treated as descriptive and interpreted with caution (Robinaugh et al., 2020). Network Comparison Tests were used to examine whether network structure and global strength differed across subgroups and across waves (van Borkulo et al., 2023).

## 3. Results

### 3.1. The Relationship Between FoMO and PSMU

The demographic characteristics of the sample are presented in Table 1. After controlling for sex and age, a partial correlation analysis revealed a positive correlation between FoMO and PSMU (*r* = 0.624, *p* < 0.001). In a hierarchical linear regression, age and gender were entered in Step 1 (*R*^2^ = 0.016), and FoMO was entered in Step 2 (Δ*R*^2^ = 0.374, F Change = 520.60, *p* < 0.001). The addition of FoMO significantly increased the explained variance in PSMU, and the final model showed that FoMO was positively associated with PSMU (*R*^2^ = 0.390, *β* = 0.617, 95% *CI* [0.566,0.672], *p* < 0.001).

**Figure 1:**
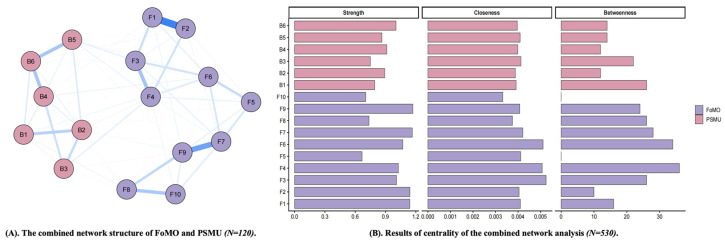
Results of the combined network structure of FoMO and PSMU. (**A**) nodes indicate items of FoMO and PSMU, and edges indicate correlations between items; thickness of edges indicate strength of correlation (BSMAS was used to assess PSMU). (**B**) the larger values, the higher centrality (BSMAS was used to assess PSMU). F1–F10 represent Items 1–10 of the FoMO scale, and B1–B6 represent Items 1–6 of the BSMAS. Blue lines indicate positive associations, whereas red lines indicate negative associations. Line thickness and color intensity indicate the strength of associations between nodes, with thicker and darker lines representing stronger associations.

The cross-sectional combined network analysis reported in this section was based on the T1 data. There were 10 items for FoMO and 6 items for PSMU, resulting in a total of 120 possible edges, of which 83 had non-zero weights (sparsity = 0.31). Positive associations were observed both within the FoMO community and within the PSMU community, and the two communities were also positively interconnected (See Figure 1).

In terms of node centrality, item 9 of FoMO (*When I miss out on a planned get-together it bothers me*) showed the highest strength, followed by item 7 of FoMO (*It bothers me when I miss an opportunity to meet up with friends*). In addition, item 3 of FoMO (*I get worried when I find out my friends are having fun without me*) showed the highest closeness. Item 4 of FoMO (*I get anxious when I don’t know what my friends are up to*) showed the highest betweenness, followed closely by item 6 of FoMO (*Sometimes, I wonder if I spend too much time keeping up with what is going on*). Because some centrality indices, especially closeness and betweenness, may be less stable in psychological networks, these findings should be interpreted descriptively rather than as definitive evidence of causal or mechanistic importance.

Bridge centrality analysis further showed that item 5 of PSMU (*Become restless or troubled if you have been prohibited from using social media?*) had the highest bridge strength in the overall network, followed by item 3 of PSMU (*Used social media in order to forget about personal problems?*), item 4 of FoMO (*I get anxious when I don’t know what my friends are up to*), and item 6 of FoMO (*Sometimes, I wonder if I spend too much time keeping up with what is going on*). Among the FoMO items, item 4 and item 6 showed the highest bridge strength, suggesting that these nodes played particularly important roles in linking the FoMO and PSMU communities. Among the PSMU items, item 5 and item 3 showed the highest bridge strength, indicating that they were the most influential PSMU nodes in transmitting associations across the two communities. Overall, these findings suggest that FoMO and PSMU were positively interconnected at the node level, with FoMO items occupying relatively central positions and several PSMU and FoMO items showing relatively high bridge strength across the two communities. These bridge-related findings are understood as candidate node-level patterns rather than definitive intervention targets.

### 3.2. The Core Nodes of FoMO and PSMU Between Different Groups

#### 3.2.1. The Core Nodes of FoMO Between Different Groups

Profile models with two to five profiles were estimated to identify the optimal FoMO solution (See Table 2). Although some information criteria continued to improve in models with more profiles, the two-profile solution was retained after considering model fit, entropy, subgroup size, and interpretability. The two-profile model showed acceptable classification quality (entropy = 0.86), with significant Lo-Mendell-Rubin and Bootstrap Likelihood Ratio Test results (LMR *p* < 0.001, BLRT *p* < 0.001). Both profiles also exceeded 5% of the total sample. Accordingly, the two-profile solution was considered the most appropriate for subsequent analyses because it provided an interpretable distinction between broadly higher and lower FoMO severity levels, whereas solutions with more profiles did not yield sufficiently clear or theoretically meaningful additional subgroup differentiation for the purposes of the present network comparisons. The retained profiles primarily differed in overall item endorsement levels rather than in qualitatively distinct response configurations and were therefore interpreted as broad severity-based groups. The first profile consisted of 344 participants and was labeled the high-FoMO group, and the second profile consisted of 509 participants and was labeled the low-FoMO group.

**Figure 2:**
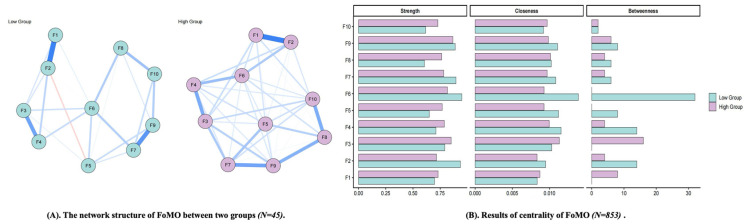
The network structure of FoMO between two groups. (**A**) nodes indicate items of FoMO, and edges indicate correlation between items; thickness of edges indicate strength of correlation. (**B**) the larger values, the higher centrality. F1–F10 represent Items 1–10 of the FoMO scale. Blue lines indicate positive associations, whereas red lines indicate negative associations. Line thickness and color intensity indicate the strength of associations between nodes, with thicker and darker lines representing stronger associations.

There were 10 FoMO items, resulting in 45 possible edges. The high-FoMO network contained 41 non-zero edges (sparsity = 0.09), whereas the low-FoMO network contained 23 non-zero edges (sparsity = 0.49). The Network Comparison Test indicated no significant difference in overall network structure (Statistic = 0.20, *p* = 0.10) or global strength (Statistic 1 = 3.42, Statistic 2 = 3.59, *T* = 0.17, *p* = 0.22) between the two networks. These results suggest that the overall organization of the FoMO network was broadly similar across the two severity-based groups (See Figure 2).

Descriptively, some variation was observed in the relative prominence of individual FoMO nodes. In the high-FoMO group, item 9 (*It bothers me when I miss out on a planned get-together*) showed the highest strength, whereas item 6 (*Sometimes, I wonder if I spend too much time keeping up with what is going on*) showed the highest closeness. In the low-FoMO group, item 6 appeared to occupy a relatively prominent position overall. Because the Network Comparison Test did not indicate significant subgroup differences in overall structure or global strength, these findings are interpreted as limited descriptive variation in node salience rather than evidence of distinct subgroup-specific network organization. Results of the detailed validation are displayed in the Supplementary Materials.

#### 3.2.2. Results of Network Analysis for PSMU Between Different Groups

Profile models with two to five profiles were estimated to identify the optimal PSMU solution (See Table 3). Although some information criteria continued to improve in models with more profiles, the two-profile solution was retained after considering model fit, entropy, subgroup size, and interpretability. The two-profile model showed acceptable classification quality (entropy = 0.88), with significant Lo-Mendell-Rubin and Bootstrap Likelihood Ratio Test results (LMR *p* < 0.001, BLRT *p* < 0.001). Both profiles also exceeded 5% of the total sample.

Accordingly, the two-profile solution was considered the most appropriate for subsequent analyses because it provided an interpretable distinction between broadly higher and lower PSMU severity levels, whereas solutions with more profiles did not yield sufficiently clear or theoretically meaningful additional subgroup differentiation for the purposes of the present network comparisons. The retained profiles likewise appeared to reflect broad differences in overall severity rather than clearly distinct qualitative subtypes. The first profile consisted of 227 participants and was labeled the high-PSMU group, and the second profile consisted of 626 participants and was labeled the low-PSMU group.

**Figure 3:**
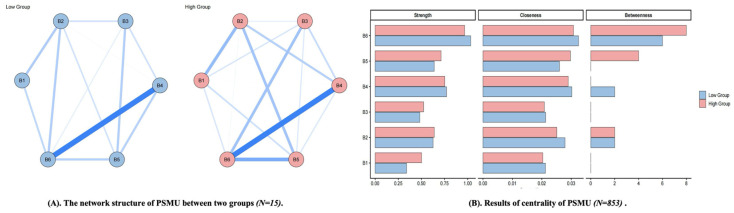
The network structure of PSMU between two groups. (**A**) nodes indicate items of PSMU, and edges indicate correlation between items; thickness of edges indicate strength of correlation. (**B**) the larger values, the higher centrality. B1–B6 represent Items 1–6 of the BSMAS. Blue lines indicate positive associations. Line thickness and color intensity indicate the strength of associations between nodes, with thicker and darker lines representing stronger associations.

There were 6 PSMU items, resulting in 15 possible edges. The high-PSMU network contained 15 non-zero edges (sparsity = 0.00), whereas the low-PSMU network contained 12 non-zero edges (sparsity = 0.20). The Network Comparison Test indicated no significant difference in overall network structure (Statistic = 0.13, *p* = 0.73) or global strength (*Statistic* 1 = 1.69, Statistic 2 = 1.80, *T* = 0.10, *p* = 0.17) between the two networks. These findings suggest that the overall organization of PSMU nodes was broadly similar across the two severity-based groups (See Figure 3).

Descriptively, item 6 of PSMU (*Negative impact on my study and work because of excessive social media use*) appeared to occupy a relatively prominent position in both the high-PSMU and low-PSMU groups. This pattern suggests that perceived functional impairment may represent a salient component of PSMU across severity levels. However, because no significant subgroup differences were found in broader network structure or global strength, the subgroup findings are better interpreted as limited descriptive variation in node prominence rather than as evidence of broader structural differences between subgroup networks. Results of the detailed validation are displayed in the Supplementary Materials.

### 3.3. The Core Nodes of FoMO and PSMU During T1 (2022) to T2 (2023)

Two-wave data were used to examine whether the relative prominence of FoMO and PSMU nodes, as well as broader network characteristics, differed from T1 (December 2022) to T2 (December 2023). Overall, the two-wave network analyses suggested greater stability than change in overall network structure, with the clearest temporal difference emerging in the global strength of the PSMU network. Any additional differences were more evident in the descriptive prominence of specific nodes than in broader network reorganization. Accordingly, node-level shifts, including bridge-node differences, should be interpreted cautiously and primarily as descriptive rather than definitive findings.

#### 3.3.1. Two-Wave Comparison of the FoMO Network

**Figure 4:**
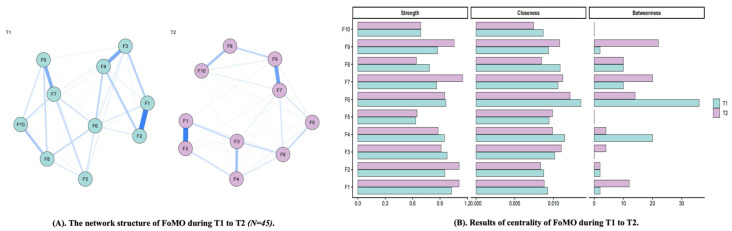
The network structure of FoMO during T1 to T2. (**A**) nodes indicate items of FoMO, and edges indicate correlation between items; thickness of edges indicate strength of correlation. (**B**) the larger values, the higher centrality. F1–F10 represent Items 1–10 of the FoMO scale. Blue lines indicate positive associations, whereas red lines indicate negative associations. Line thickness and color intensity indicate the strength of associations between nodes, with thicker and darker lines representing stronger associations.

There were 10 FoMO items, resulting in 45 possible edges. The T1 network contained 33 non-zero edges (sparsity = 0.27), whereas the T2 network contained 36 non-zero edges (sparsity = 0.20). The Network Comparison Test indicated no significant difference in overall network structure between T1 and T2 (*M* = 0.13, *p* = 0.25) and no significant difference in global strength (*T1* = 4.24, *T2* = 4.35, *S* = 0.11, *p* = 0.20). These findings suggest that the overall configuration and total connectivity of the FoMO network remained broadly stable across the two waves, with only limited descriptive variation in the relative prominence of specific nodes (See Figure 4).

Specifically, in T1, item 1 of FoMO (*I fear others have more rewarding experiences than me*) showed the highest strength, and item 3 of FoMO (*I get worried when I find out my friends are having fun without me*) showed the second-highest strength, indicating that these two items were the central nodes in the FoMO network at T1. In addition, item 6 of FoMO (*Sometimes, I wonder if I spend too much time keeping up with what is going on*) showed the highest closeness and betweenness in T1. In T2, item 7 of FoMO (*It bothers me when I miss an opportunity to meet up with friends*) showed the highest strength, item 6 of FoMO (*Sometimes, I wonder if I spend too much time keeping up with what is going on*) showed the highest closeness, and item 9 of FoMO (*When I miss out on a planned get-together it bothers me*) showed the highest betweenness. These findings suggest limited descriptive variation in the relative prominence of FoMO nodes across waves. Because the Network Comparison Test did not indicate significant two-wave differences in overall network structure or global strength, these node-level shifts should be interpreted cautiously and should not be taken as evidence of broader network reorganization. Results of the detailed validation are displayed in the Supplementary Materials.

#### 3.3.2. Two-Wave Comparison of the PSMU Network

**Figure 5:**
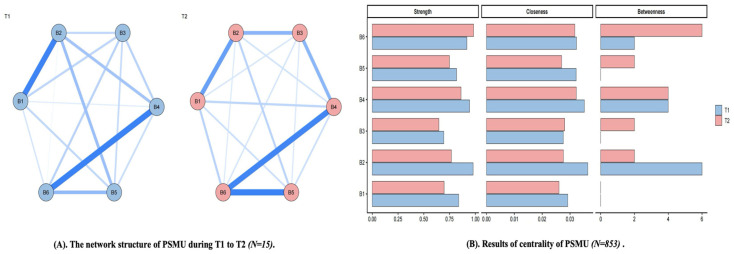
The network structure of PSMU during T1 to T2. (**A**) nodes indicate items of PSMU, and edges indicate correlation between items; thickness of edges indicate strength of correlation. (**B**) the larger values, the higher centrality. B1–B6 represent Items 1–6 of the BSMAS. Blue lines indicate positive associations. Line thickness and color intensity indicate the strength of associations between nodes, with thicker and darker lines representing stronger associations.

There were six PSMU items, resulting in 15 possible edges at both T1 and T2. The Network Comparison Test indicated that the overall network structure did not differ significantly between T1 and T2 (*M* = 0.15, *p* = 0.074). However, the global strength invariance test was significant. Specifically, global strength was 2.52 at T1 and 2.24 at T2, and the global strength invariance test was significant (*S* = 0.28, *p* < 0.001). These findings suggest that although the overall arrangement of PSMU nodes remained broadly stable across time, the overall intensity of associations among PSMU nodes changed significantly (See Figure 5).

Specifically, at T1, item 2 of PSMU (*Felt an urge to use social media more and more?*) showed the highest strength, closeness, and betweenness, indicating that it was the most central node in the T1 PSMU network. At T2, item 6 of PSMU (*Used social media so much that it has had a negative impact on your job/studies?*) showed the highest strength and betweenness, whereas item 4 (*Tried to cut down on the use of social media without success?*) showed the highest closeness. This pattern suggests that item 6 occupied a relatively prominent position in the T2 network, although different nodes played distinct roles across centrality indices. Results of the detailed validation are displayed in Supplementary Materials.

#### 3.3.3. Two-Wave Comparison of the Combined FoMO-PSMU Network

**Figure 6:**
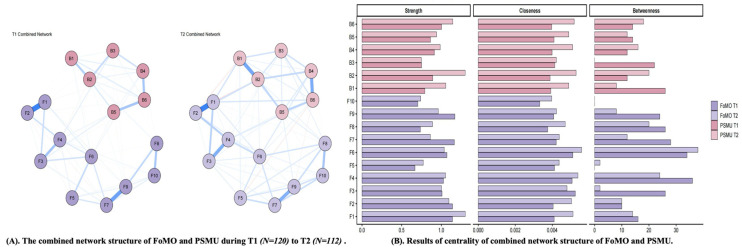
The combined network structure of FoMO and PSMU during T1 to T2. (**A**) nodes indicate items of FoMO and PSMU, and edges indicate correlation between items, while thickness of edges indicate strength of correlation (BSMAS was used to assess PSMU). (**B**) the larger values, the higher centrality. F1–F10 represent Items 1–10 of the FoMO scale, and B1–B6 represent Items 1–6 of the BSMAS. Blue lines indicate positive associations, whereas red lines indicate negative associations. Line thickness and color intensity indicate the strength of associations between nodes, with thicker and darker lines representing stronger associations.

For the combined FoMO–PSMU network, 16 items yielded 120 possible edges. The T1 combined network contained 83 non-zero edges (sparsity = 0.31), whereas the T2 combined network contained 80 non-zero edges (sparsity = 0.33). The Network Comparison Test indicated no significant difference in overall network structure (*M* = 0.14, *p* = 0.226) and no significant difference in global strength between T1 and T2. Specifically, global strength was 6.85 at T1 and 7.06 at T2, and the global strength invariance test was not significant (*S* = 0.21, *p* = 0.074). These findings indicate that the relative prominence of several bridging nodes varied descriptively across the two waves, although the combined FoMO-PSMU network did not show clear evidence of broader structural reorganization over time (See Figure 6).

Specifically, in T1, item 6 of FoMO (*Sometimes I get confused because I spend too much time trying to find out what is going on*) showed the strongest bridge strength among the FoMO items, whereas item 5 of PSMU showed the highest bridge strength in the overall combined network. In addition, item 4 of FoMO (*I get anxious when I don’t know what my friends are up to*) also showed relatively high bridge strength in T1. This pattern suggests that, at T1, FoMO items related to uncertainty and anxiety about others’ activities were particularly important in connecting the FoMO and PSMU communities. In T2, item 6 of FoMO again showed relatively high bridge strength among the FoMO items, but the highest bridge strength in the overall combined network was observed for item 2 of PSMU, followed by item 6 of PSMU. Among the FoMO items, item 1 and item 8 also showed relatively high bridge strength in T2. These findings indicate that the most influential bridging nodes differed across time, and that the bridge structure of the combined FoMO–PSMU network was not dominated by a single FoMO node at both waves. Because the broader combined-network comparisons were non-significant, these bridge-node differences are best understood as preliminary and descriptive rather than as definitive evidence of structural transformation. Results of the detailed validation are displayed in Supplementary Materials.

## 4. Discussion

This study examined the association between FoMO and PSMU and explored their node-level organization using complementary LPA and network analysis. Three main findings emerged. First, FoMO was positively associated with PSMU at both the scale level and the node-network level. Second, most subgroup and two-wave network comparisons suggested greater stability than difference in overall network structure and global strength. Third, when variation did emerge, it was more evident in the relative prominence of specific nodes, including several bridge-related nodes, than in broader network organization. Taken together, the findings suggest that the FoMO-PSMU association is robust and that subgroup- and time-related differences are better understood as limited node-level variation within a broader pattern of structural stability.

### 4.1. Association Between FoMO and PSMU

The present findings suggest that FoMO was positively associated with PSMU. Both the correlation and regression analyses indicated that individuals with higher FoMO tended to report higher levels of problematic social media use. This association was also reflected at the node level, as the combined network analysis showed positive interconnections between FoMO and PSMU nodes. Taken together, these findings provide converging evidence that FoMO and PSMU are closely related in this sample, which is consistent with previous research reporting a robust positive association between the two constructs (Akbari et al., 2021; Montag et al., 2023; Servidio et al., 2025). At the same time, previous studies have more often established the presence of an association than clarified whether that association reflects stable co-occurrence patterns at the node level, directional influence, or subgroup-specific organization.

One possible interpretation is that concerns about missing out on others’ rewarding experiences may be linked to stronger tendencies to engage with social media in maladaptive ways. This interpretation is broadly consistent with the I-PACE model, which emphasizes the role of cognitive-affective processes in the development and maintenance of problematic online behaviors (Brand et al., 2019). It is also in line with prior work suggesting that FoMO may heighten sensitivity to socially relevant cues and reinforce repeated checking or engagement tendencies in online environments (Przybylski et al., 2013; Wegmann et al., 2017). At the same time, the present findings should be interpreted cautiously. Although FoMO and PSMU were positively connected at both the scale and node levels, the current design does not permit causal conclusions about whether FoMO contributes to PSMU, whether PSMU intensifies FoMO, or whether both processes occur simultaneously over time (Brand et al., 2019; Robinaugh et al., 2020). Accordingly, the most appropriate conclusion is that FoMO and PSMU showed a robust positive association in this sample, rather than that the present findings establish a directional mechanism between them.

### 4.2. Differences in Core Nodes Across Severity-Based Subgroups

The subgroup analyses suggested limited variation in the relative prominence of specific nodes across severity-based FoMO and PSMU groups, whereas most broader network characteristics remained stable. For FoMO, the Network Comparison Test did not reveal significant differences in overall network structure or global strength between the high- and low-FoMO groups. This finding suggests that the overall organization of FoMO nodes was broadly similar across subgroup levels, even though some individual nodes appeared relatively more prominent in one group than in the other (van Borkulo et al., 2023). Descriptively, item 9 and item 6 appeared relatively more prominent in the high-FoMO group, whereas item 6 appeared to occupy a comparatively prominent position in the low-FoMO group. One possible interpretation is that when FoMO is more severe, concerns about missing planned social experiences may become relatively more salient, whereas cognitive preoccupation with staying informed may remain important across different levels of FoMO. This interpretation is consistent with previous work suggesting that FoMO includes both social-comparative and cognitively preoccupying components (Akbari et al., 2021; Wang et al., 2022). However, because subgroup differences were not significant at the level of overall network structure or global strength, these observations are better understood as tentative differences in node salience rather than evidence of clearly distinct FoMO network organizations.

For PSMU, the subgroup comparison likewise did not reveal significant differences in overall network structure or global strength between the high- and low-PSMU groups. This suggests that the general organization of PSMU nodes may be relatively similar across severity-based subgroups (van Borkulo et al., 2023). Descriptively, item 6, reflecting negative impact on study and work due to excessive social media use, appeared relatively prominent in both groups. This pattern may indicate that perceived functional impairment represents a salient aspect of problematic social media use across severity levels, rather than only in more severe presentations. Such an interpretation is broadly consistent with prior research characterizing problematic social media use in terms of continued engagement despite adverse consequences and functional difficulties (Brand et al., 2019; Fournier et al., 2023; Shannon et al., 2022). At the same time, because no significant subgroup differences were found in broader network structure, the PSMU findings should also be interpreted cautiously and primarily as limited descriptive variation in node prominence rather than as evidence of distinct subgroup-specific node structures.

Taken together, the subgroup findings suggest that severity-based FoMO and PSMU groups may differ modestly in the relative salience of specific nodes, but they do not support a strong conclusion that subgroup networks differ in their broader structural organization. In this sense, the present results point more toward relative stability in overall network architecture, accompanied by limited node-level variation, than toward qualitatively distinct subgroup-specific networks.

### 4.3. Two-Wave Differences in Node Prominence and Network Connectivity

The two-wave analyses suggested that FoMO and PSMU networks were characterized more by overall stability than by marked structural change. This is an important finding, because it suggests that the broader node architecture linking FoMO and PSMU may remain relatively stable over a one-year period even when the salience of individual nodes fluctuates. In other words, node-level variation and structural stability are not contradictory; rather, they may coexist within the same broader system. This distinction is important for interpreting longitudinal network findings more broadly.

For FoMO, no significant difference was observed in either network structure or global strength across the two waves, indicating that any temporal variation was limited and more evident in the relative prominence of specific nodes than in broader network organization (Robinaugh et al., 2020; van Borkulo et al., 2023). More specifically, T1 was characterized by relatively prominent FoMO nodes involving concerns about others’ rewarding experiences, exclusion-related worry, and cognitive preoccupation, whereas T2 showed relatively greater prominence for nodes related to missed social opportunities, cognitive preoccupation, and distress about missing planned gatherings. One possible interpretation is that FoMO-related concerns may shift modestly in emphasis over time (Akbari et al., 2021; Wang et al., 2022) while remaining embedded within a stable broader network organization. Because the Network Comparison Test did not indicate significant differences in overall network structure or global strength for FoMO across the two waves (van Borkulo et al., 2023), these node-level shifts are better interpreted as limited variation in node salience rather than broader network reorganization.

For PSMU, the overall network structure also remained broadly stable, although the global strength comparison suggested a significant change in the overall intensity of node associations (van Borkulo et al., 2023). More specifically, the most prominent node appeared to shift from item 2, reflecting a stronger urge to use social media, at T1 to item 6, reflecting negative impact on study and work due to excessive social media use, at T2. This pattern may suggest that motivational aspects of social media use are relatively more salient at one stage, whereas awareness of functional consequences may become more salient at another. However, because no significant difference was found in overall network structure, the two-wave PSMU findings are better interpreted as reflecting a change in the overall intensity of associations among nodes, alongside limited variation in node prominence, rather than a clear reorganization of the network structure. Taken together, these two-wave findings suggest greater stability than change in the FoMO and PSMU networks, with any differences being more evident at the level of relative node prominence and global connectivity than at the level of broader structural organization.

The combined FoMO-PSMU network did not show significant differences in either overall structure or global strength across the two waves. Therefore, the two-wave combined-network findings are best interpreted as reflecting descriptive variation in the relative prominence of specific nodes, particularly bridging nodes, rather than changes in overall network connectedness or clear structural transformation. One possible implication is that FoMO and PSMU remained closely linked across waves, while the relative salience of particular bridging nodes varied over time (L. Li et al., 2021; Brand et al., 2019). However, because the broader network comparisons were non-significant, these bridge-node differences should be regarded as preliminary and descriptive rather than as definitive evidence of temporal reorganization in the combined network.

Although the present findings should not be overinterpreted as identifying definitive intervention targets, they may still have preliminary practical relevance. In university settings, students with elevated FoMO may represent a psychologically meaningful risk group for PSMU. In addition, nodes related to social uncertainty, repeated checking, loss of control, and perceived functional impairment may be useful candidate targets for prevention or early intervention efforts, pending further longitudinal and experimental validation.

### 4.4. Limitations and Future Research

Several limitations should be acknowledged. First, the sample consisted of Chinese university students recruited through convenience-based online dissemination, which may limit the generalizability of the findings to other age groups, educational settings, or cultural contexts (Akbari et al., 2021; Shannon et al., 2022). Future research should examine whether similar FoMO–PSMU patterns can be replicated in more diverse populations. Second, all variables were assessed using self-report questionnaires, which may have introduced recall bias, social desirability bias, or shared method variance. In addition, although two-wave data were available, the present analyses focused primarily on between-wave network comparisons rather than models of temporal or directional influence. Accordingly, conclusions about longitudinal dynamics should remain cautious, and future studies could use cross-lagged approaches or related longitudinal models to examine temporal relations between FoMO and PSMU more directly (Brand et al., 2019; Robinaugh et al., 2020). Third, most subgroup and two-wave network comparisons did not reveal significant differences in overall structure, which means that the observed variation in node prominence should be interpreted as preliminary and primarily descriptive. Relatedly, closeness and betweenness were interpreted cautiously because these indices may be less stable and less readily interpretable in psychological networks (Robinaugh et al., 2020). Fourth, the retained two-profile solutions may reflect broad severity-based distinctions rather than qualitatively distinct latent subtypes. At the measurement level, the one-factor FoMO scale may not fully capture the multidimensional nature of FoMO. Previous work has suggested that general FoMO and online-specific FoMO may represent related but distinguishable constructs (Wegmann et al., 2017). Likewise, although differential prominence of individual items within a network framework does not by itself invalidate the use of total scores at the construct level, it does suggest that FoMO and PSMU may show heterogeneous internal organization at the item level. In addition, although the Bergen Social Media Addiction Scale is widely used in research on problematic social media use (Luo et al., 2021), some authors have noted that scale-based screening instruments may not fully align with stricter addiction frameworks or diagnostic formulations (Fournier et al., 2023). Future research would therefore benefit from multimethod, clinically informed, and more explicit psychometric and longitudinal approaches when examining the relationship between FoMO and PSMU.

## 5. Conclusions

This study showed that FoMO was positively associated with PSMU among Chinese university students and extended this conclusion by examining the relationship using complementary latent profile and two-wave network comparison approaches. Across subgroup and two-wave analyses, the FoMO-PSMU relationship appeared relatively stable at the overall network level, with differences emerging more clearly in the relative prominence of specific nodes than in broader network structure. These findings contribute to a more cautious and fine-grained understanding of the FoMO-PSMU relationship by suggesting that subgroup- and time-related variation may be better conceptualized as limited node-level fluctuation within a broader pattern of structural stability. Future research should test these preliminary node-level patterns using stronger longitudinal, multimethod, and clinically informed designs.

## Figures and Tables

**Table 1:** Demographic characteristics of the sample.

Demographic Information		N (%)	Mean (SD)	Max	Min
gender	male	350 (41.0%)			
	female	503 (59.0%)			
age		853	20.72 (2.4)	36	18
FoMO		853	33.37 (7.7)	50	10
BSMAS		853	20.43 (5.3)	30	6

FoMO: the Chinese version of the FoMO scale. BSMAS: the Chinese version of the Bergen Social Media Addiction Scale.

**Table 2:** Results of LPA of FoMO.

Model	AIC	BIC	aBIC	LMR	BLRT	Entropy	Latent Class
2	24,405.030	24,552.242	24,453.795	<0.001	<0.001	0.861	0.40328/0.59672
3	23,726.571	23,926.019	23,792.639	0.0056	<0.001	0.849	0.24502/0.45604/0.29894
4	23,265.400	23,517.084	23,348.772	<0.001	<0.001	0.857	0.21336/0.38218/0.13482/0.13482
5	21,928.468	22,229.629	22,026.390	0.0027	<0.001	0.857	0.08201/0.24113/0.25704/0.17258/0.24725

**Table 3:** Results of LPA of PSMU.

Model	AIC	BIC	aBIC	LMR	BLRT	Entropy	Latent Class
2	13,759.532	13,849.759	13,789.420	<0.001	<0.001	0.876	0.26612/0.73388
3	13,199.339	13,322.806	13,240.238	0.0006	<0.001	0.898	0.21571/0.63658/0.14771
4	12,994.699	13,151.408	13,046.609	0.0283	<0.001	0.797	0.17351/0.27667/0.42204/0.12778
5	13,600.264	13,790.215	13,663.186	0.0443	<0.001	0.798	0.11606/0.12544/0.23798/0.41032/0.11020

## Data Availability

The original contributions presented in this study are included in the article/Supplementary Materials. Further inquiries can be directed to the corresponding authors.

## Supplementary Materials

The following supporting information can be downloaded at: https://www.mdpi.com/article/10.3390/bs16050678/s1, Figure S1. Edge-weight accuracy for FoMO and PSMU integrated symptoms network in the total sample. Bootstrapped confidence intervals of estimated edge-weights for the estimated network. The red line indicates the sample values, and the gray area indicates the bootstrapped confidence intervals. Each horizontal line represents one edge of the network, ordered from the edge with the highest edge-weight to the edge with the lowest edge-weight. Figure S2. Centrality stability for FoMO and PSMU integrated symptoms network in the total sample. Average correlations between centrality indices of networks sampled with persons dropped and the original sample. Lines indicate the means and areas indicate the range from the 2.5th quantile to the 97.5th quantile (BSMAS represent the level of PSMU). Figure S3. Node strength centrality difference test for FoMO and PSMU integrated symptoms network in the total sample. The gray box represents nodes or edges that do not differ significantly from one-another, the black box represents nodes or edges that do differ significantly from one-another, and the white box in the centrality plot shows the value of node strength (BSMAS represent the level of PSMU). Figure S4. Edge-weight accuracy for FoMO symptoms network in high group. Figure S5. Centrality stability for FoMO symptoms network in high group. Figure S6. Node strength centrality difference test for FoMO symptoms network in high group. Figure S7. Edge-weight accuracy for FoMO symptoms network in low group. Figure S8. Centrality stability for FoMO symptoms network in low group. Figure S9. Node strength centrality difference test for FoMO symptoms network in low group. Figure S10. Edge-weight accuracy for PSMU symptoms network in high group. Figure S11. Centrality stability for PSMU symptoms network in high group. Figure S12. Node strength centrality difference test for PSMU symptoms network in high group. Figure S13. Edge-weight accuracy for PSMU symptoms network in low group. Figure S14. Centrality stability for PSMU symptoms network in low group. Figure S15. Node strength centrality difference test for PSMU symptoms network in low group. Figure S16. Edge-weight accuracy for FoMO symptoms network in T1. Figure S17. Centrality stability for FoMO symptoms network in T1. Figure S18. Node strength centrality difference test for FoMO symptoms network in T1. Figure S19. Edge-weight accuracy for FoMO symptoms network in T2. Figure S20. Centrality stability for FoMO symptoms network in T2. Figure S21. Node strength centrality difference test for FoMO symptoms network in T2. Figure S22. Edge-weight accuracy for PSMU symptoms network in T1. Figure S23. Centrality stability for PSMU symptoms network in T1. Figure S24. Node strength centrality difference test for PSMU symptoms network in T1. Figure S25. Edge-weight accuracy for PSMU symptoms network in T2. Figure S26. Centrality stability for PSMU symptoms network in T2. Figure S27. Node strength centrality difference test for PSMU symptoms network in T2. Figure S28. Edge-weight accuracy for combined network in T1. Figure S29. Centrality stability for combined network in T1. Figure S30. Node strength centrality difference test for combined network in T1. Figure S31. Edge-weight accuracy for combined network in T2. Figure S32. Centrality stability for combined network in T2. Figure S33. Node strength centrality difference test for combined network in T2.

## Abbreviations

The following abbreviations are used in this manuscript:

FoMO	Fear of missing out
PSMU	Problematic social media use
NCT	Network Comparison Test
LPA	Latent profile analysis
