# Supplementary material for: Fear of Missing Out and Problematic Social Media Use Among Chinese University Students: Latent Profiles and Two-Wave Network Comparisons
Source: Behav Sci (Basel). 2026 Apr 29;16(5):678. doi: 10.3390/bs16050678 (PMC13203285; doi:10.3390/bs16050678)
Supplement: Supplementary file 1 [file behavsci-16-00678-s001.zip › behavsci-4257285-supplementary.pdf]

Supplementary Material

1 Results of stability and accuracy of the combined network

The results of the combined network weights show a moderate accurate and a good node stability of the merged network.

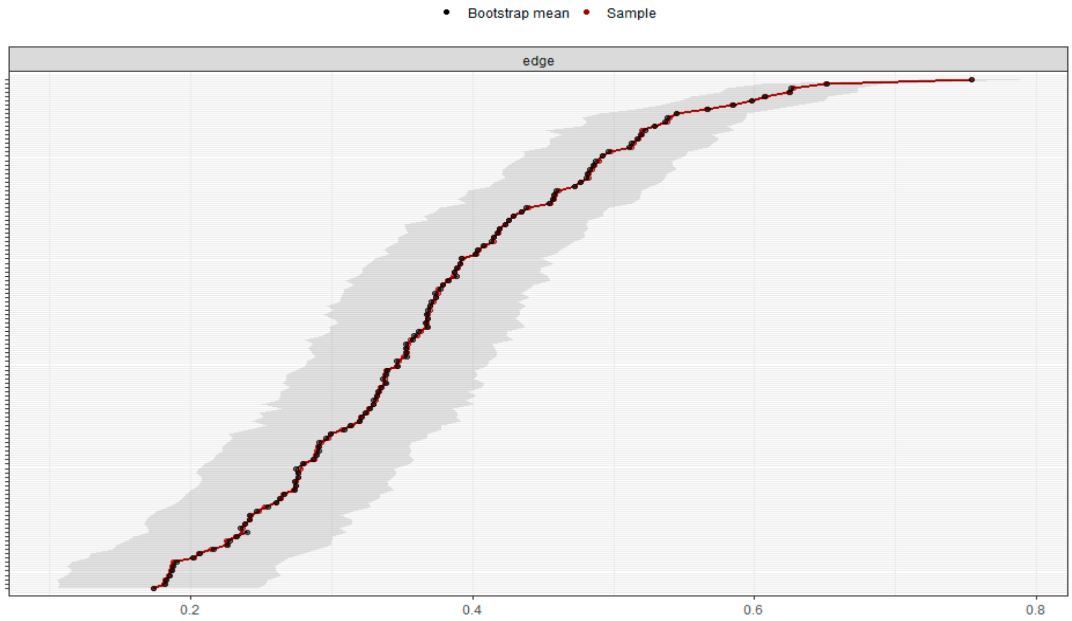

Figure S1. Edge-weight accuracy for FoMO and PSMU integrated symptoms network in the total sample. Bootstrapped confidence intervals of estimated edge-weights for the estimated network. The red line indicates the sample values, and the gray area indicates the bootstrapped confidence intervals. Each horizontal line represents one edge of the network, ordered from the edge with the highest edge-weight to the edge with the lowest edge-weight.

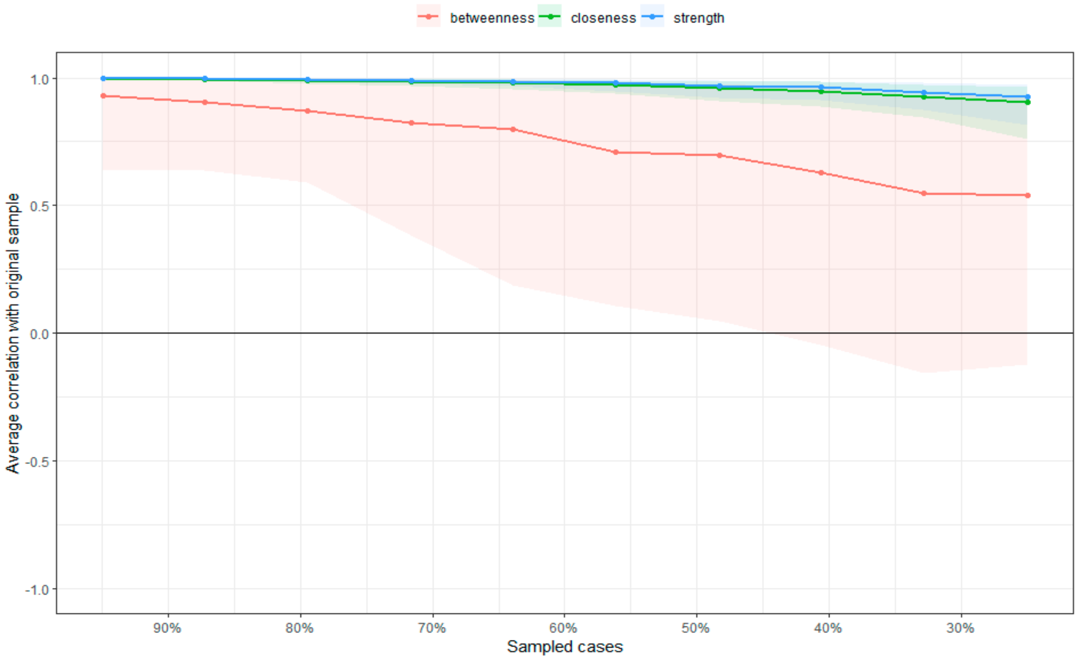

Figure S2. Centrality stability for FoMO and PSMU integrated symptoms network in the total sample. Average correlations between centrality indices of networks sampled with persons dropped and the original sample. Lines indicate the means and areas indicate the range from the 2.5th quantile to the 97.5th quantile (BSMAS represent the level of PSMU)

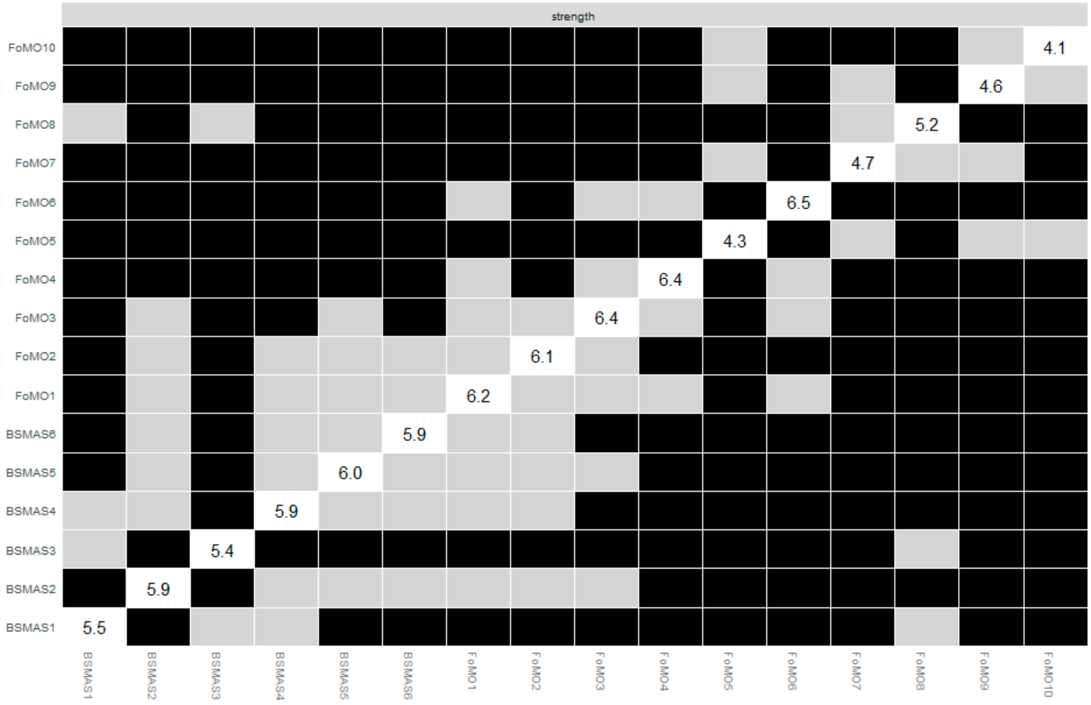

Figure S3. Node strength centrality difference test for FoMO and PSMU integrated symptoms network in the total sample. The gray box represents nodes or edges that do not differ significantly from one-another, the black box represents nodes or edges that do differ significantly from one-another, and the white box in the centrality plot shows the value of node strength (BSMAS represent the level of PSMU)

2 Results of stability and accuracy of FoMO network between two groups

The results of FoMO network show a moderate accurate and a good node stability between two groups.

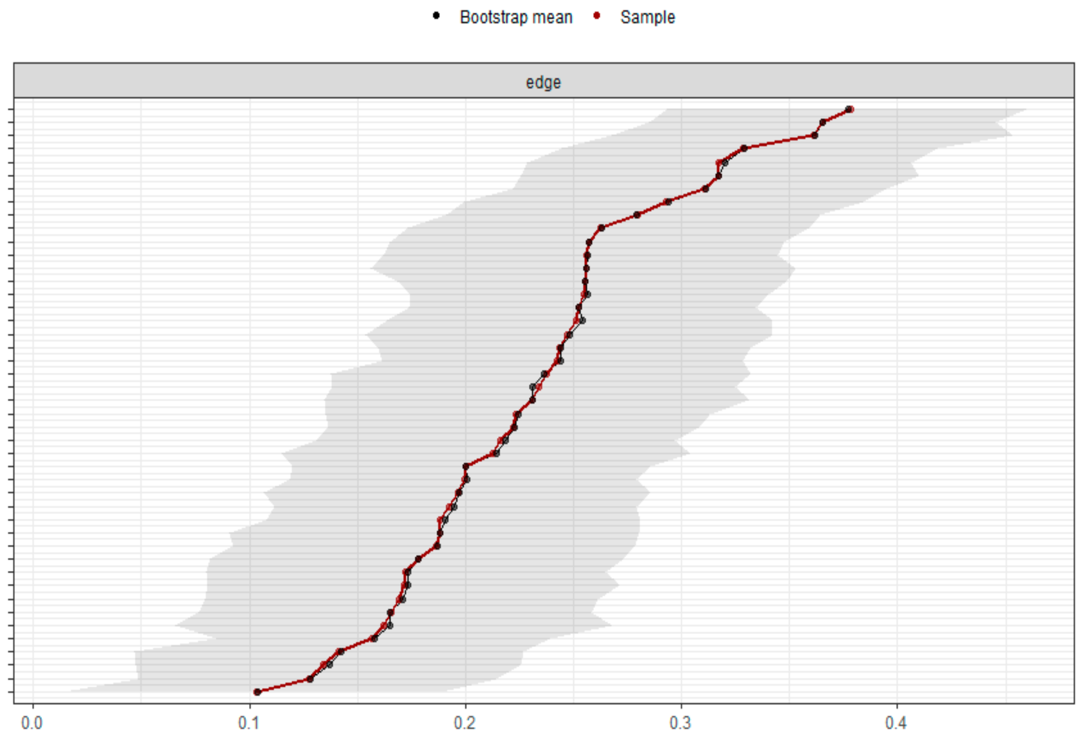

Figure S4. Edge-weight accuracy for FoMO symptoms network in high group.

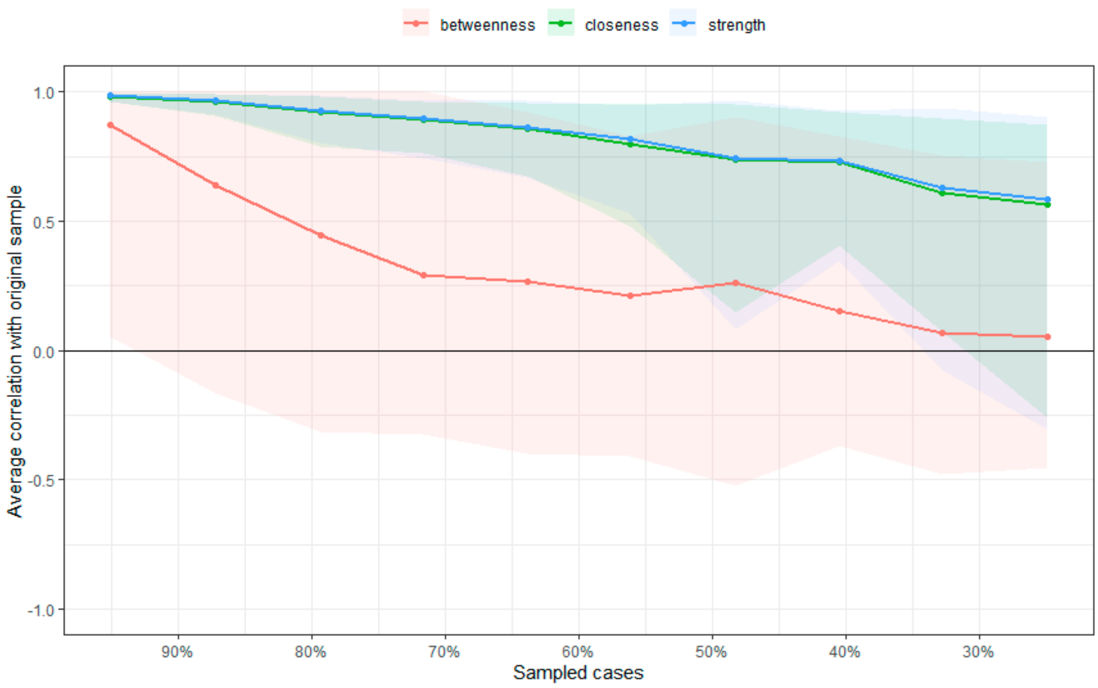

Figure S5. Centrality stability for FoMO symptoms network in high group.

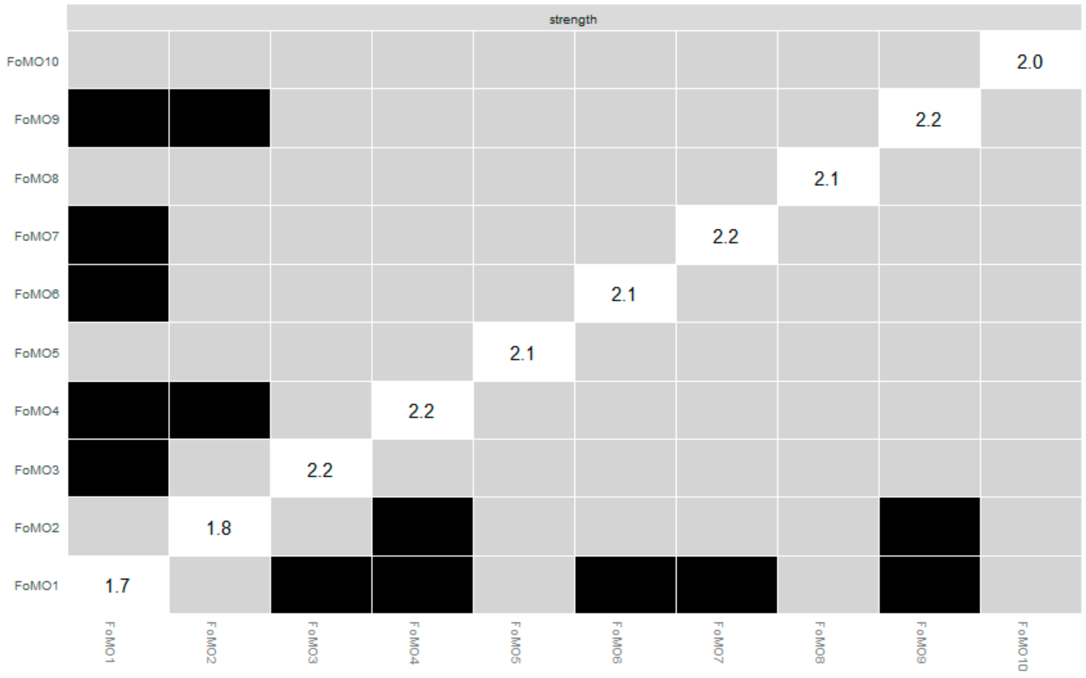

Figure S6. Node strength centrality difference test for FoMO symptoms network in high group.

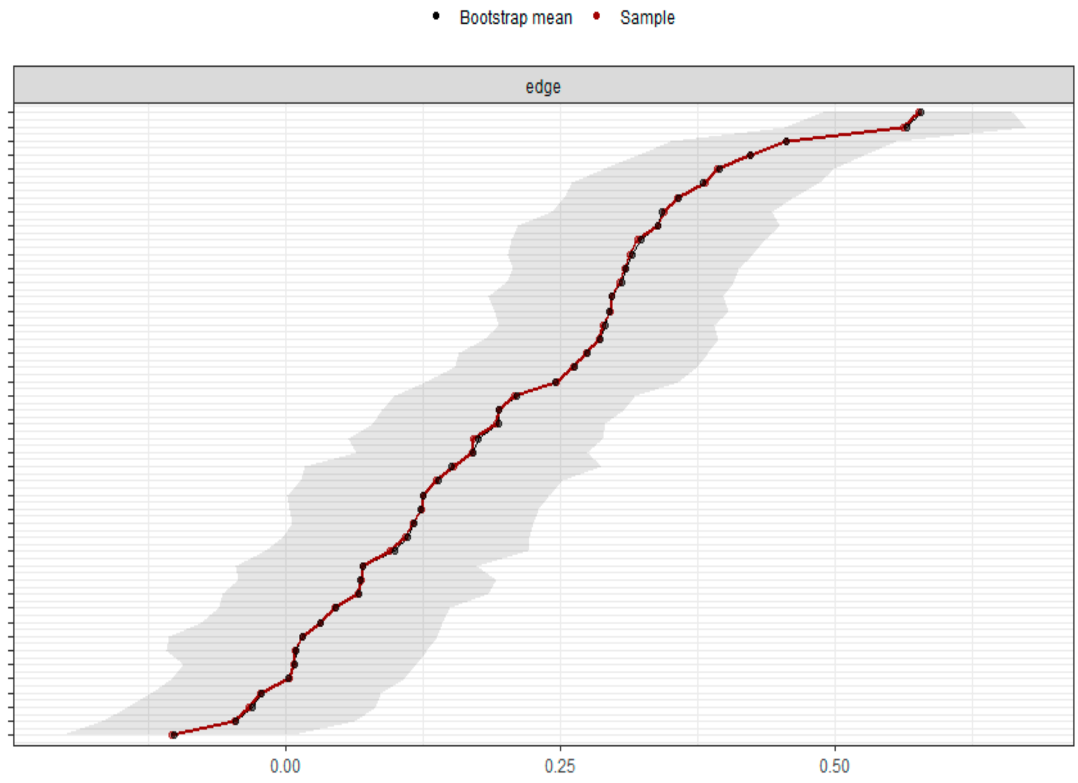

Figure S7. Edge-weight accuracy for FoMO symptoms network in low group.

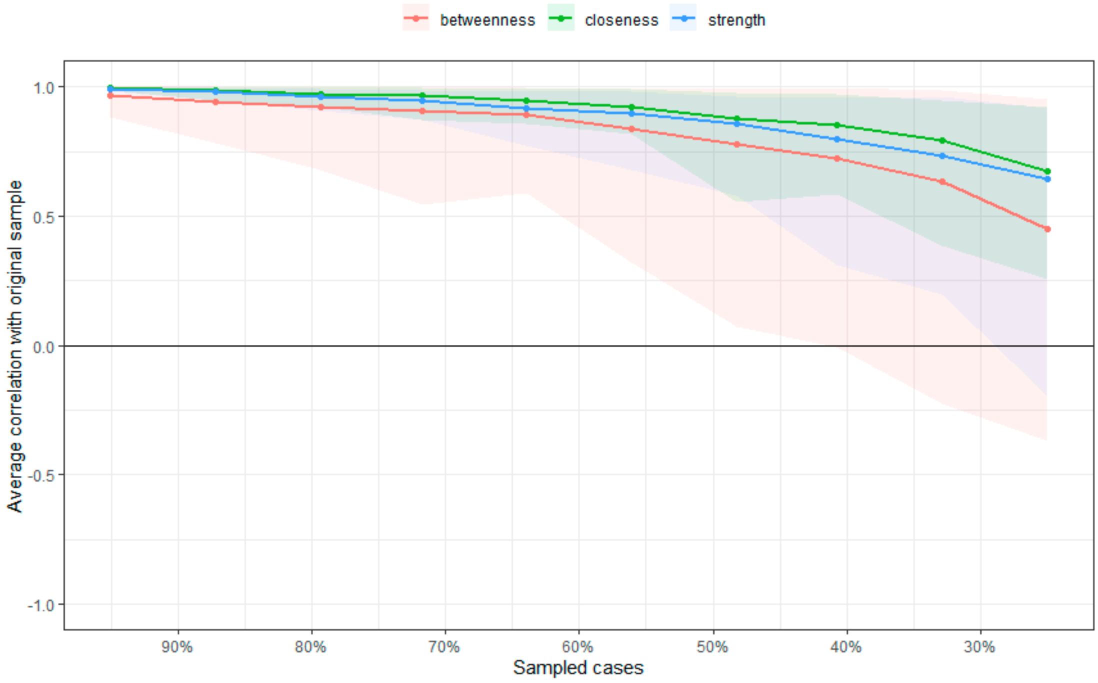

Figure S8. Centrality stability for FoMO symptoms network in low group.

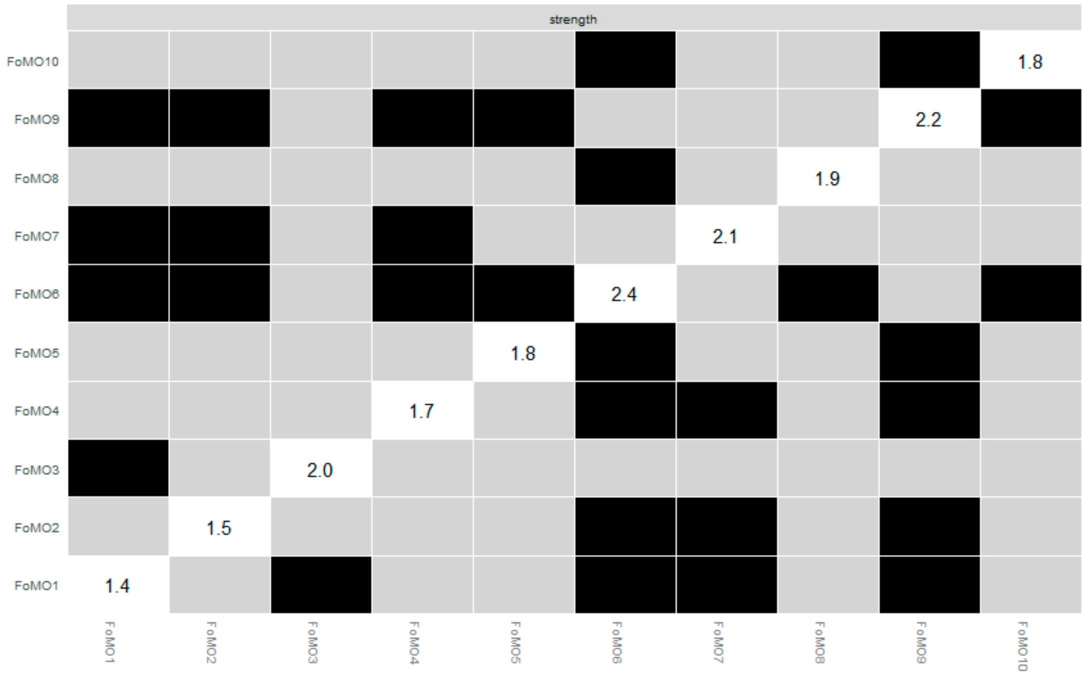

Figure S9. Node strength centrality difference test for FoMO symptoms network in low group.

3 Results of stability and accuracy of PSMU network between two groups

The results of PSMU network show a moderate accurate and a good node stability between two groups.

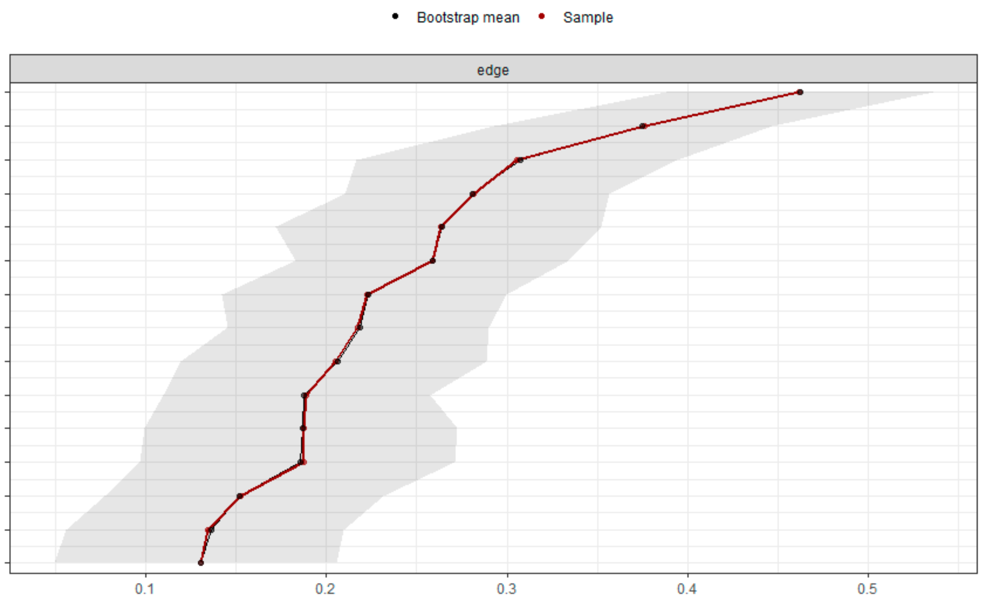

Figure S10. Edge-weight accuracy for PSMU symptoms network in high group.

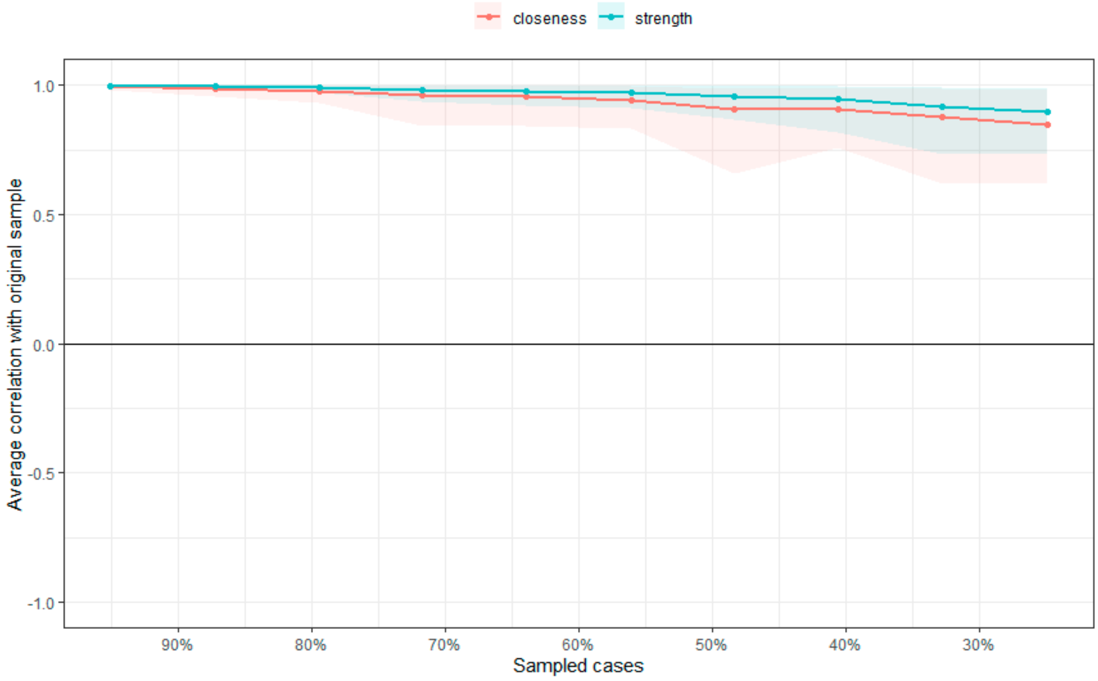

Figure S11. Centrality stability for PSMU symptoms network in high group.

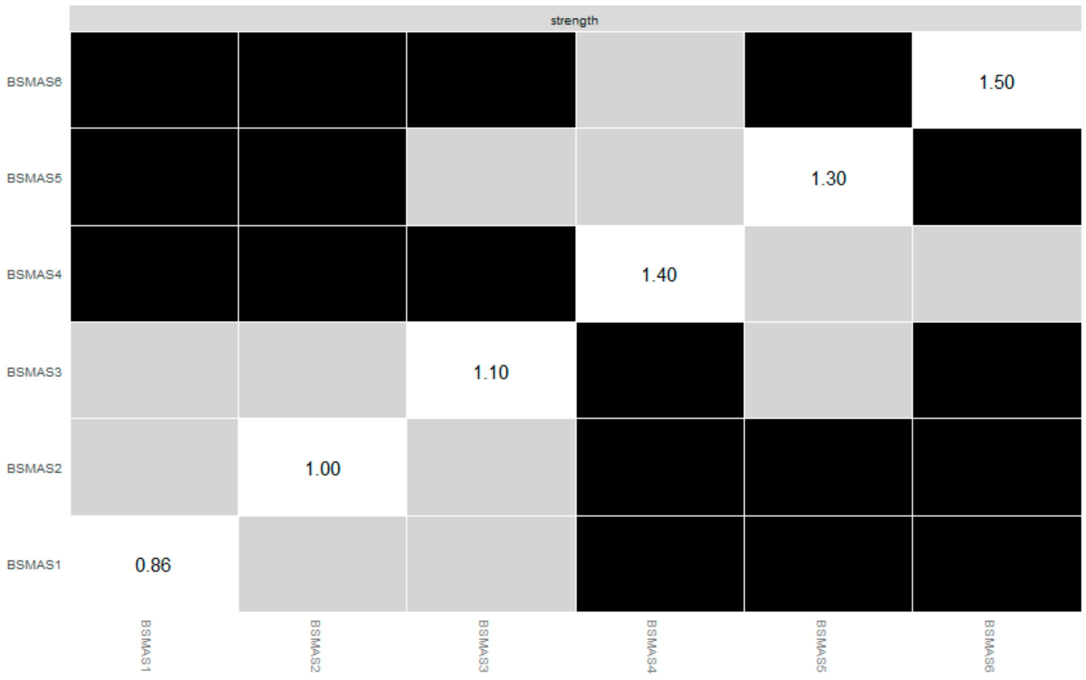

Figure S12. Node strength centrality difference test for PSMU symptoms network in high group.

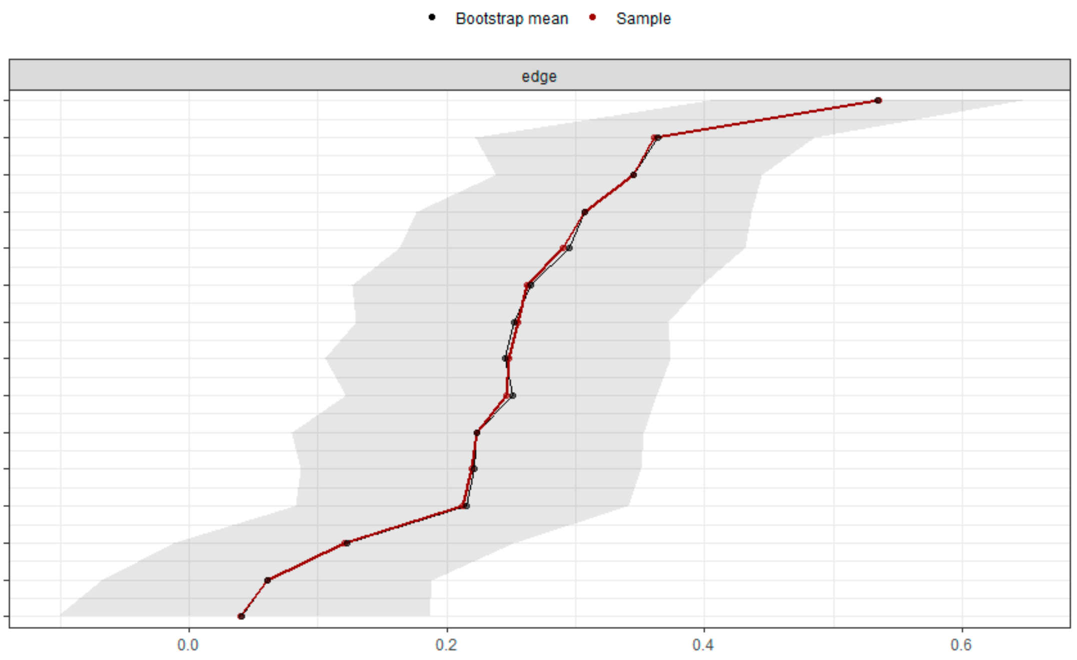

Figure S13. Edge-weight accuracy for PSMU symptoms network in low group

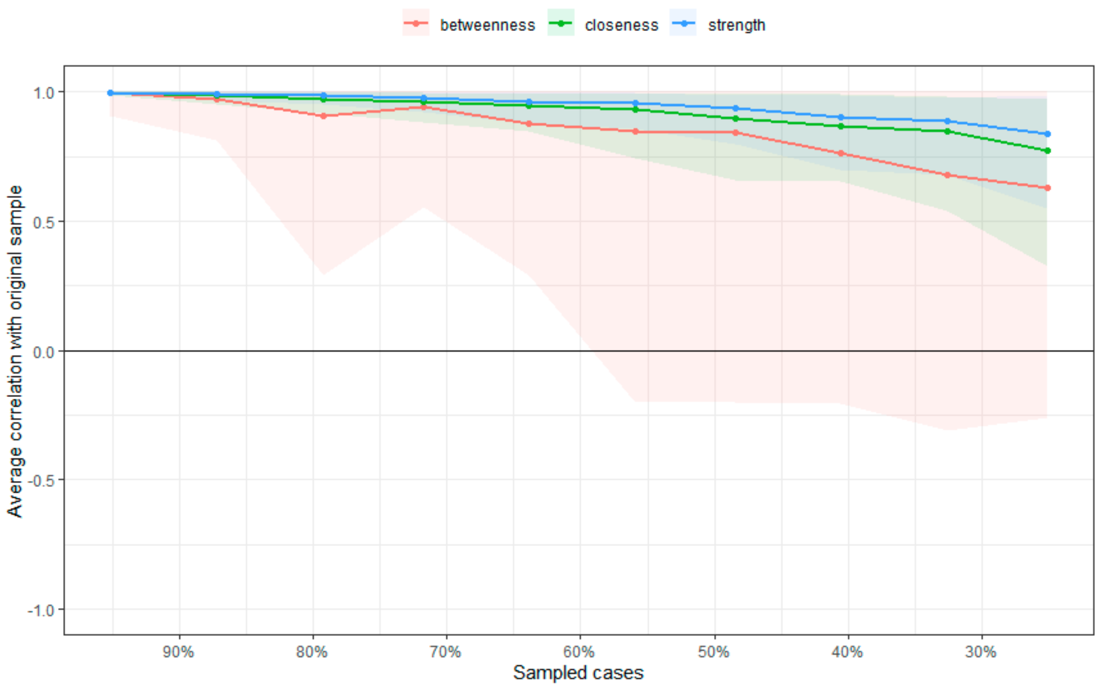

Figure S14. Centrality stability for PSMU symptoms network in low group.

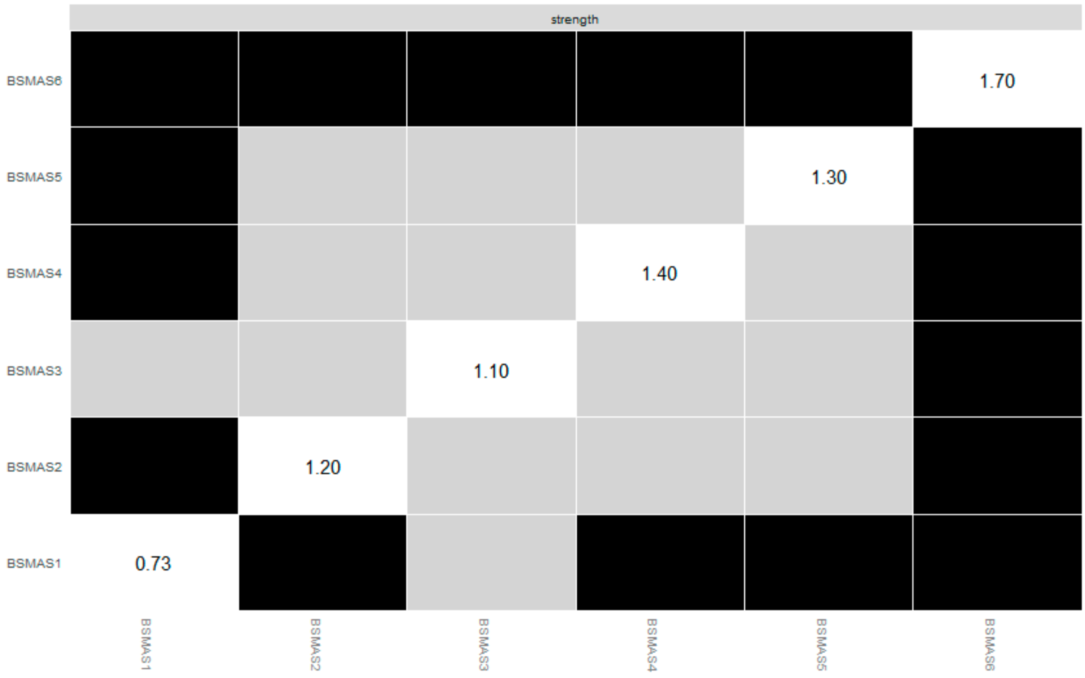

Figure S15. Node strength centrality difference test for PSMU symptoms network in low group.

4 Results of stability and accuracy of networks during T1 to T2

4.1 Results of stability and accuracy of FoMO network during T1 to T2

The results of FoMO network show a moderate accurate and a good node stability during T1 to T2.

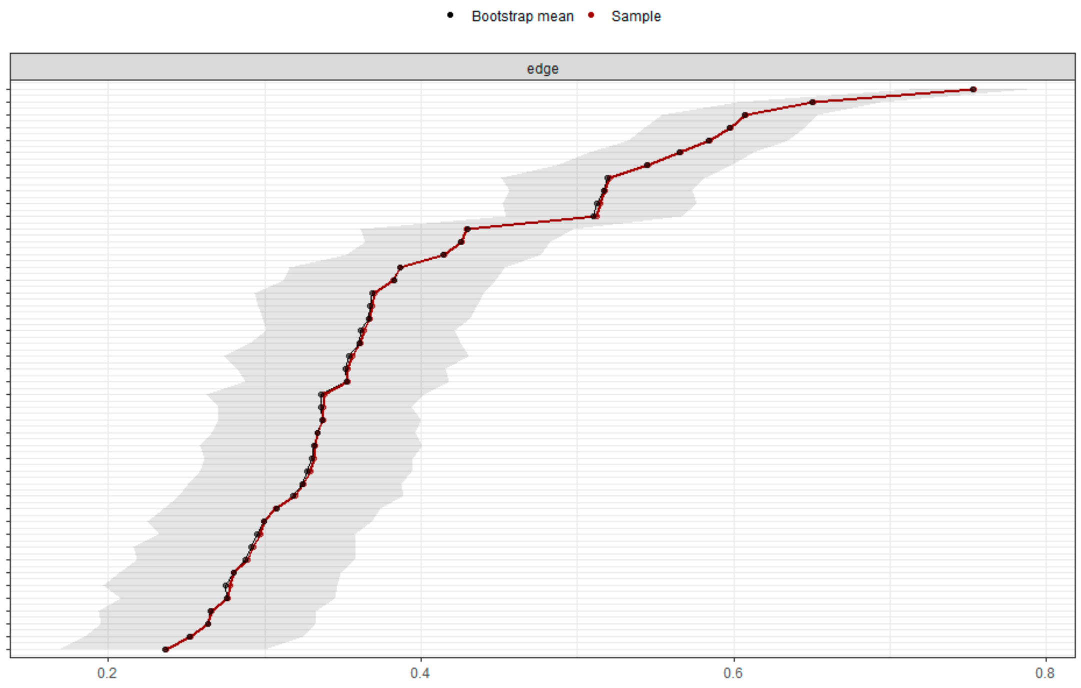

Figure S16. Edge-weight accuracy for FoMO symptoms network in T1.

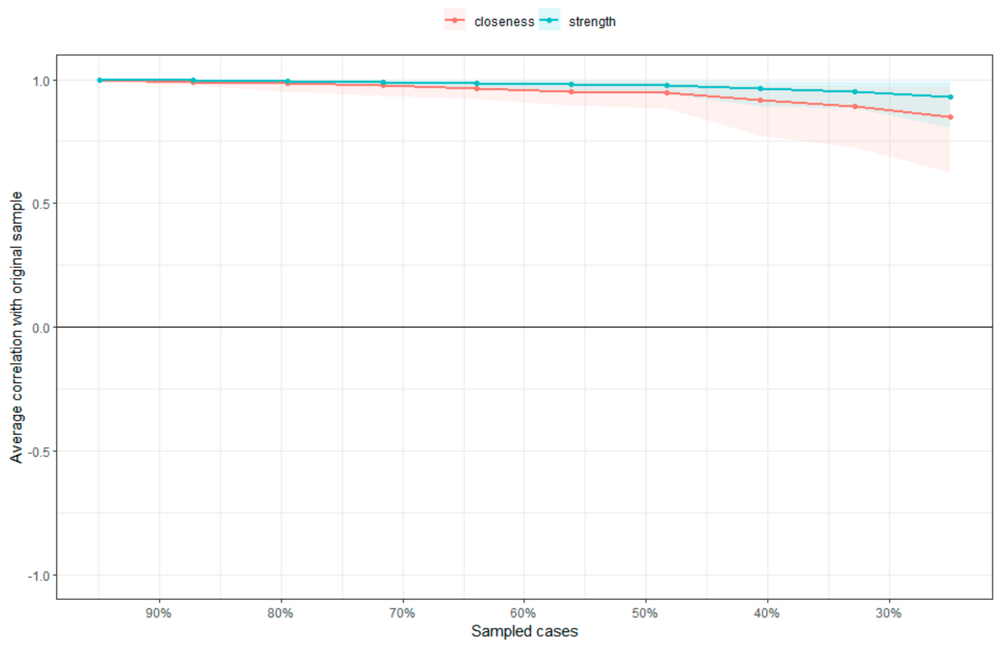

Figure S17. Centrality stability for FoMO symptoms network in T1.

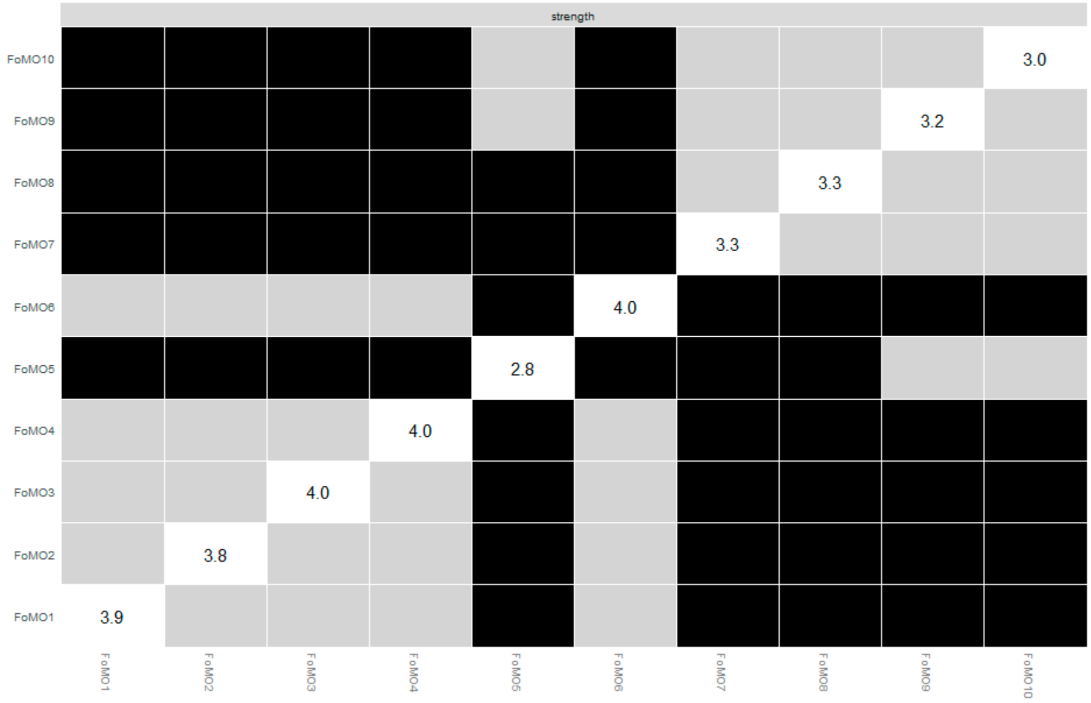

Figure S18. Node strength centrality difference test for FoMO symptoms network in T1.

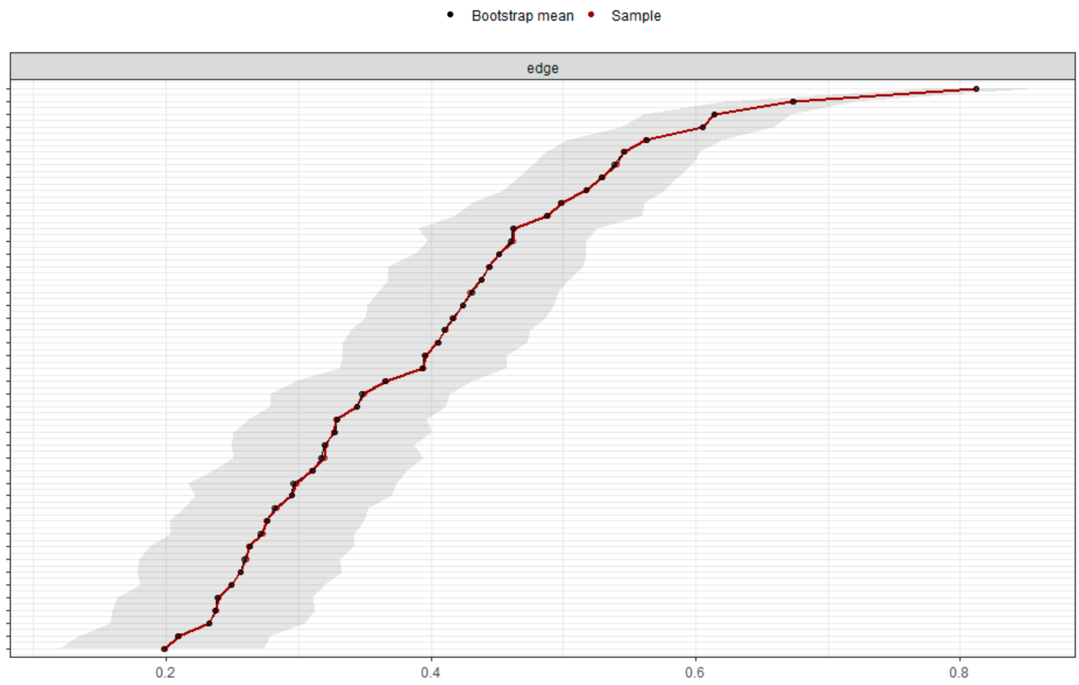

Figure S19. Edge-weight accuracy for FoMO symptoms network in T2.

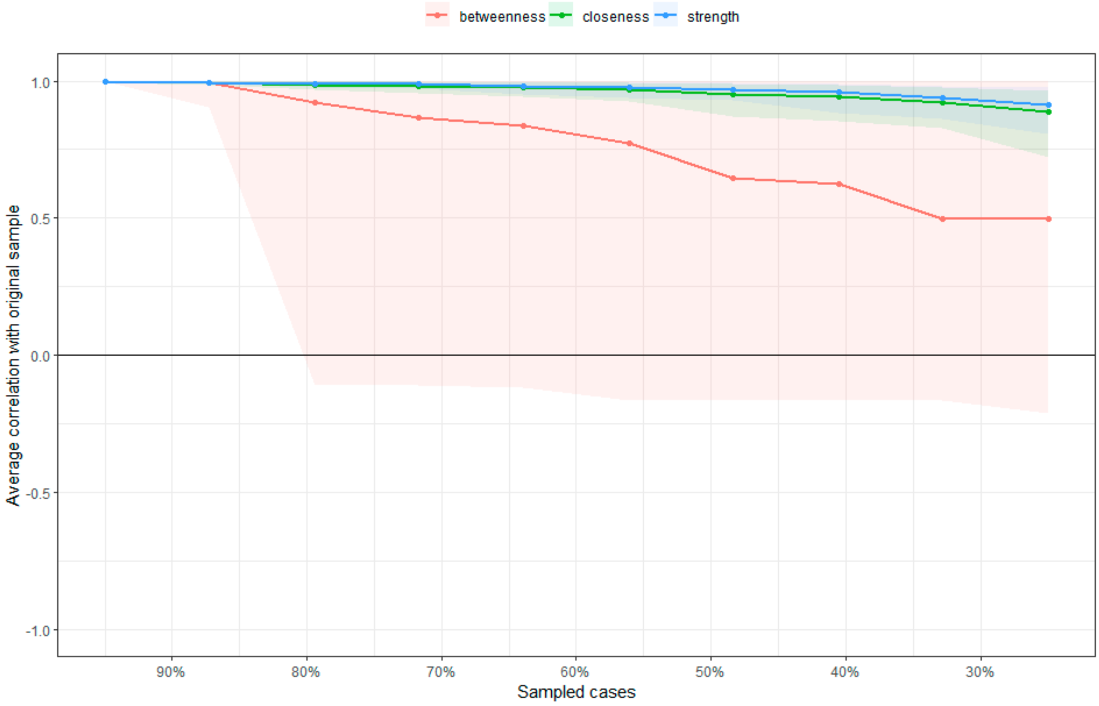

Figure S20. Centrality stability for FoMO symptoms network in T2.

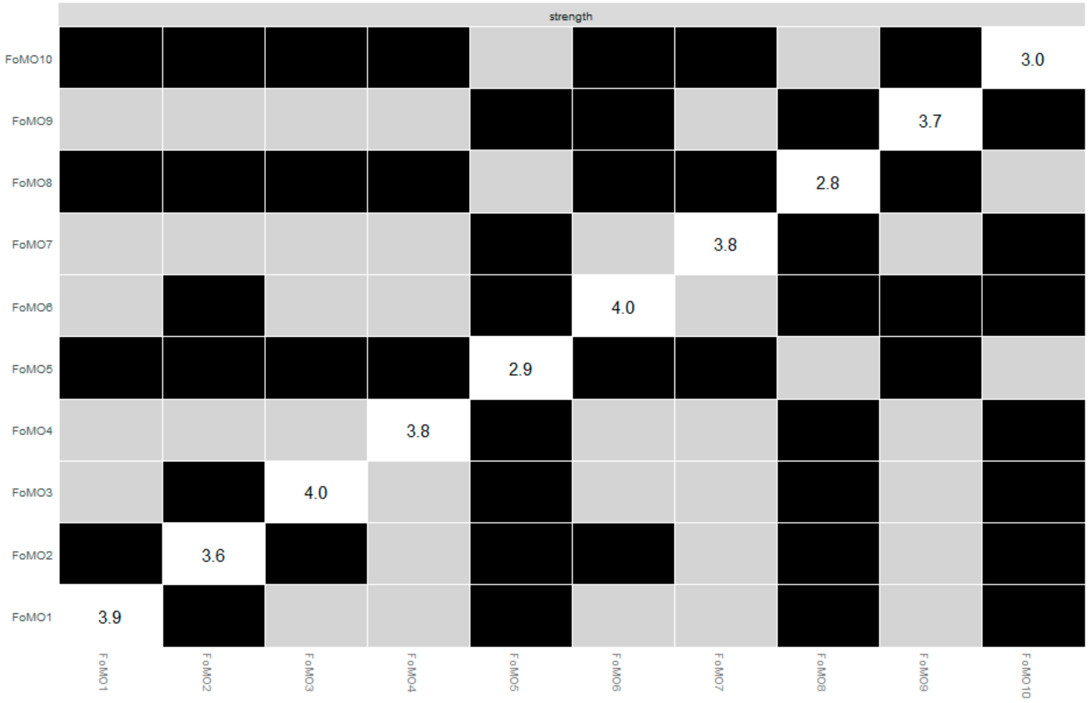

Figure S21. Node strength centrality difference test for FoMO symptoms network in T2.

4.2 Results of stability and accuracy of PSMU network during T1 to T2

The results of PSMU network show a moderate accurate and a good node stability during T1 to T2.

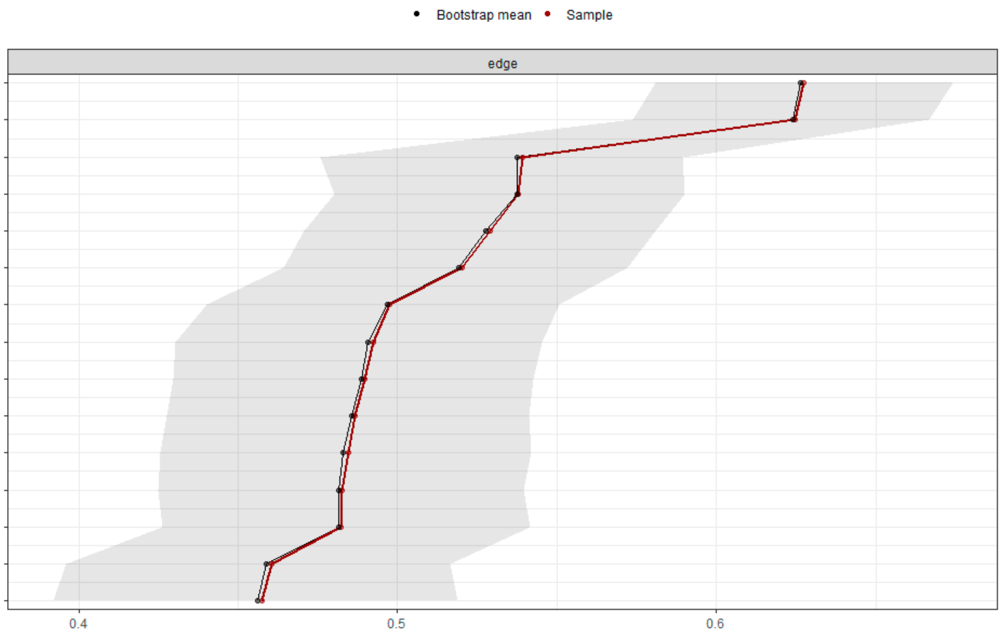

Figure S22. Edge-weight accuracy for PSMU symptoms network in T1.

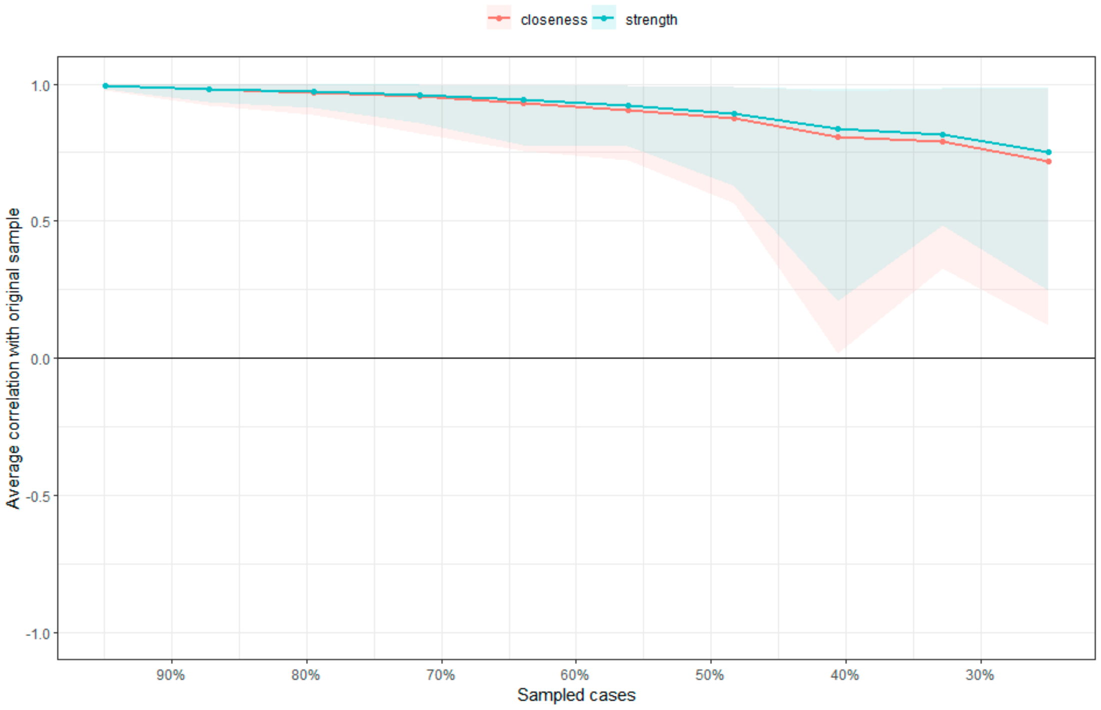

Figure S23. Centrality stability for PSMU symptoms network in T1.

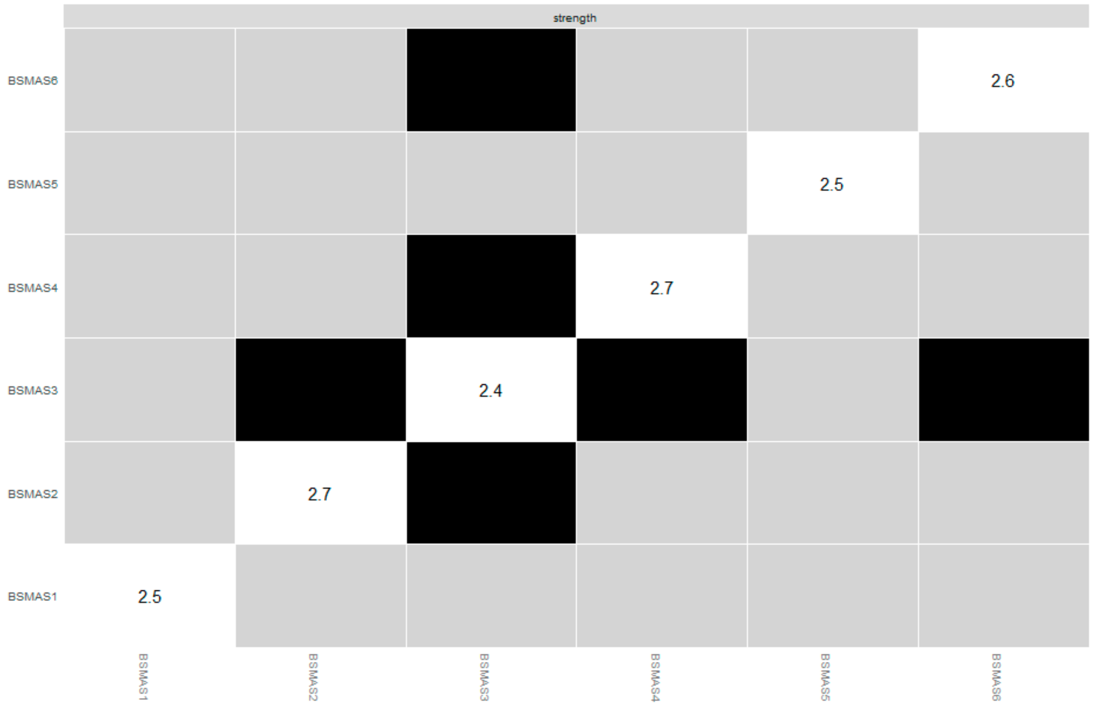

Figure S24. Node strength centrality difference test for PSMU symptoms network in T1.

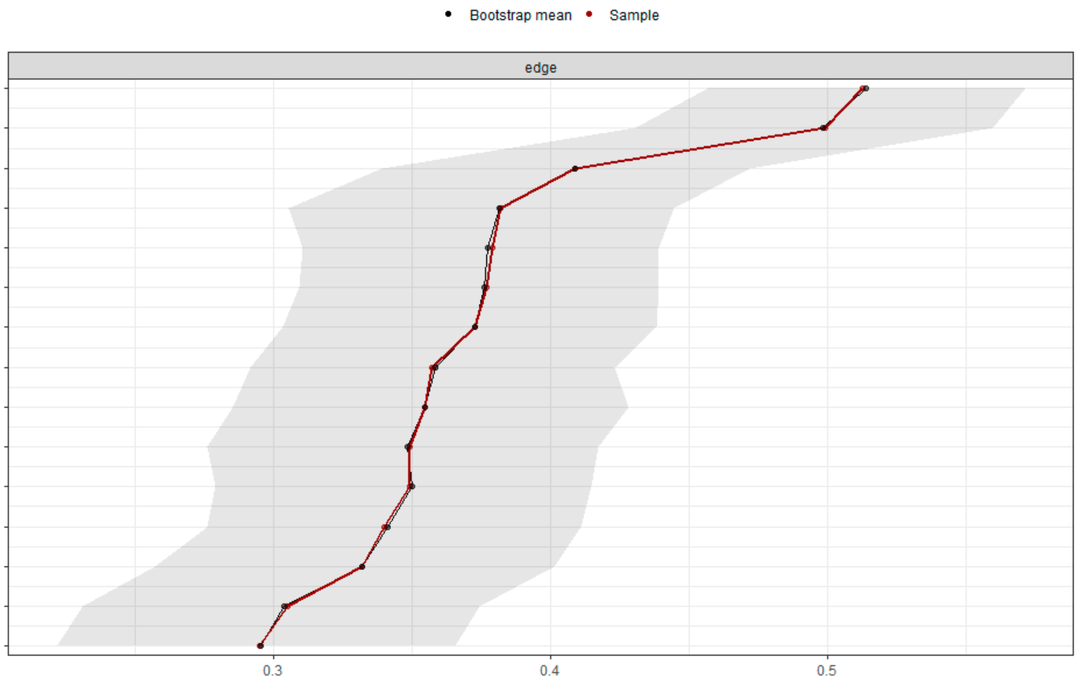

Figure S25. Edge-weight accuracy for PSMU symptoms network in T2.

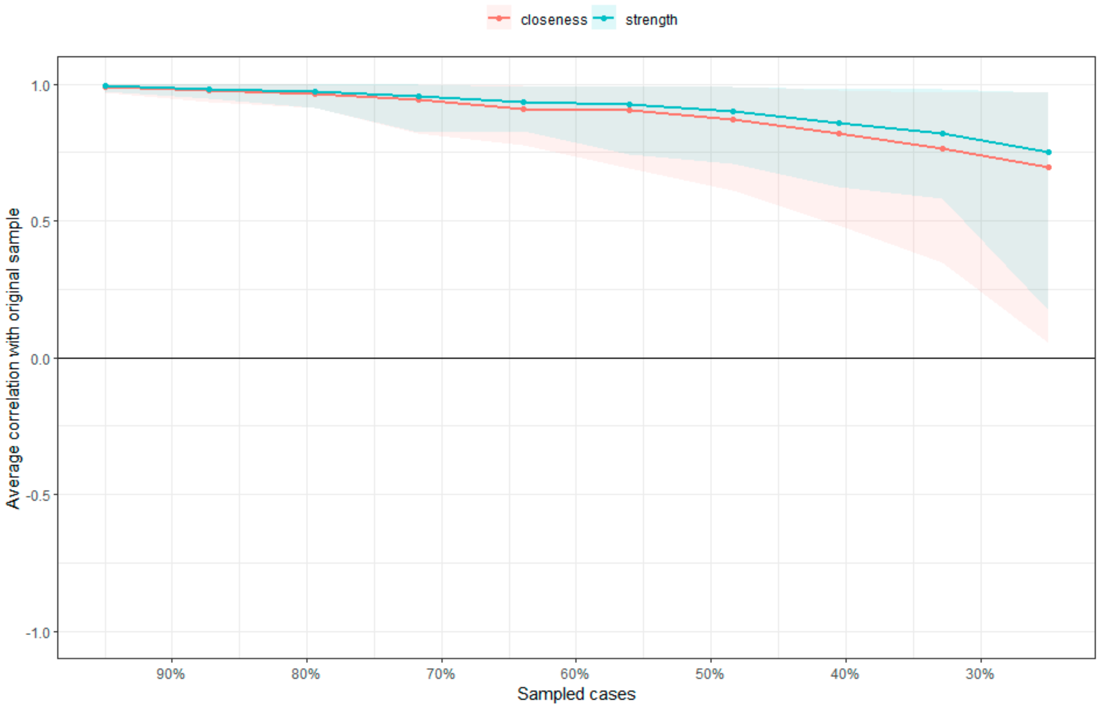

Figure S26. Centrality stability for PSMU symptoms network in T2.

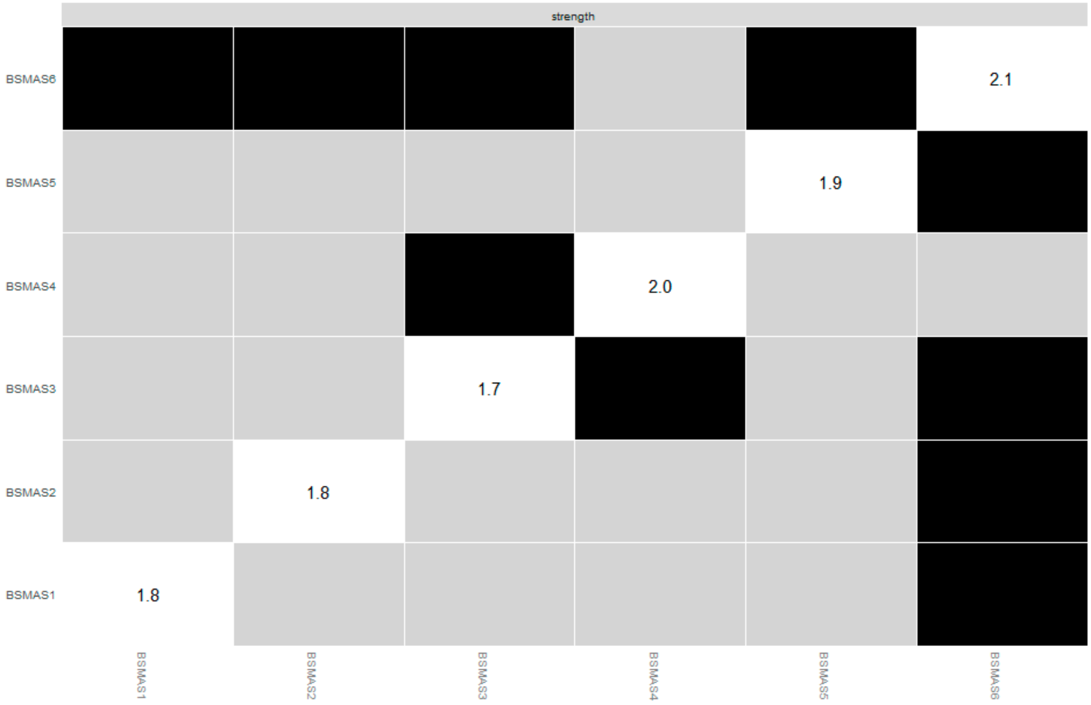

Figure S27. Node strength centrality difference test for PSMU symptoms network in T2.

4.3 Results of stability and accuracy of combined network during T1 to T2

The results of combined network show a moderate accurate and a good node stability during T1 to T2.

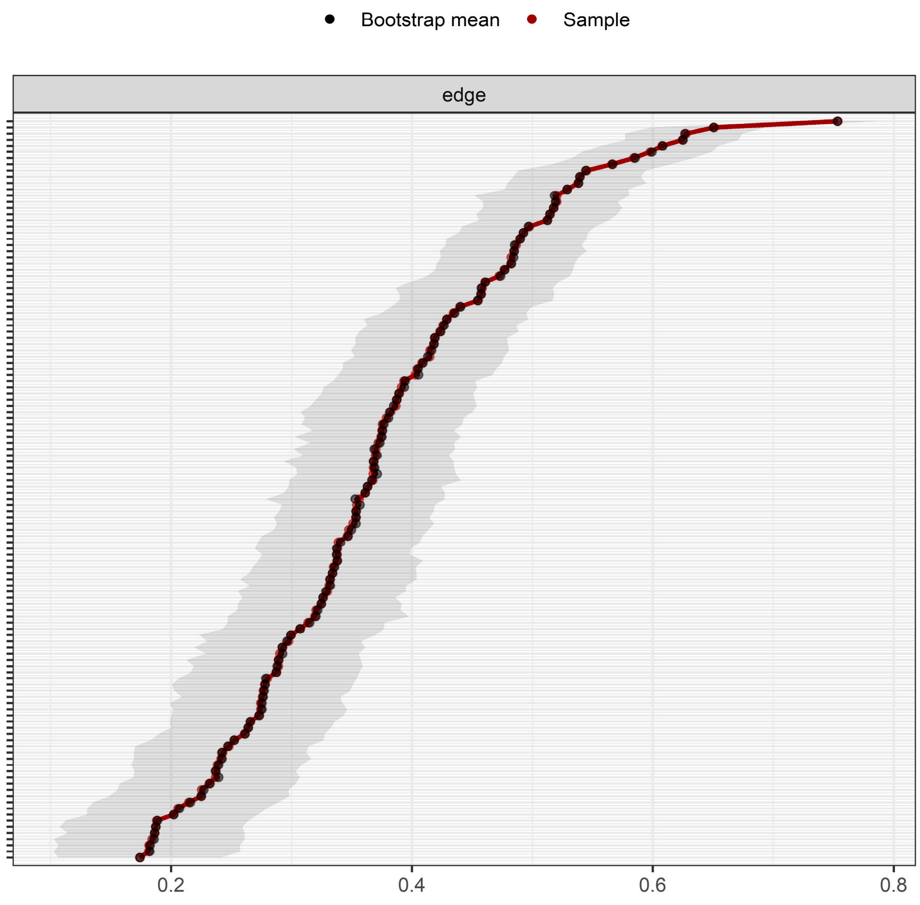

Figure S28. Edge-weight accuracy for combined network in T1.

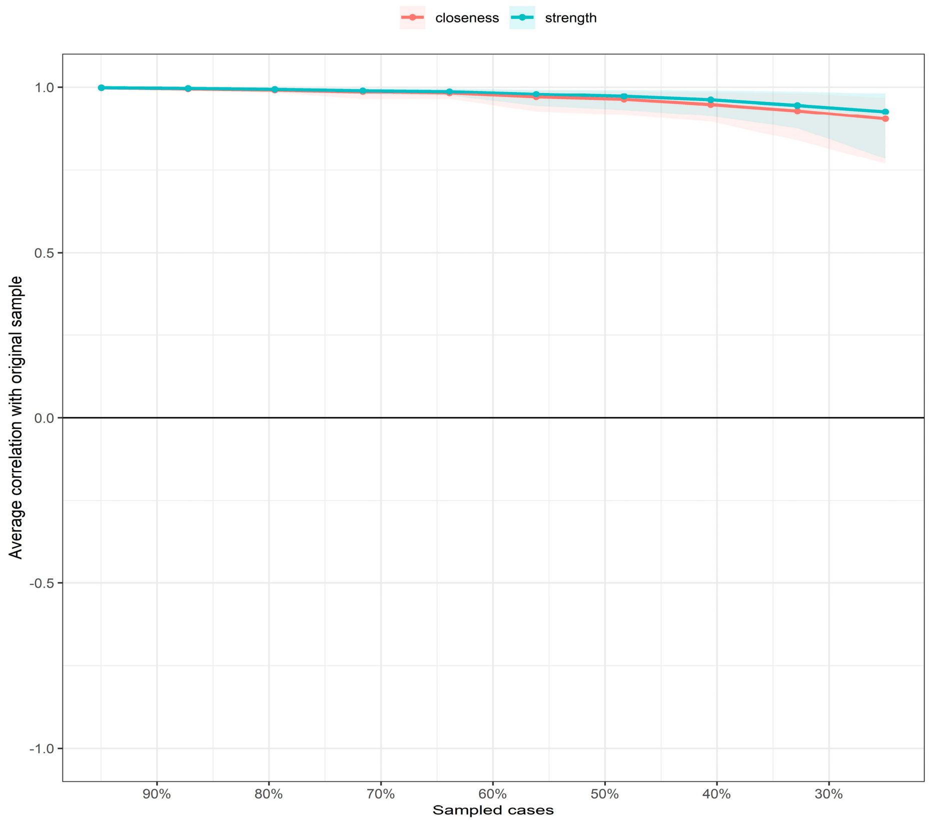

Figure S29. Centrality stability for combined network in T1.

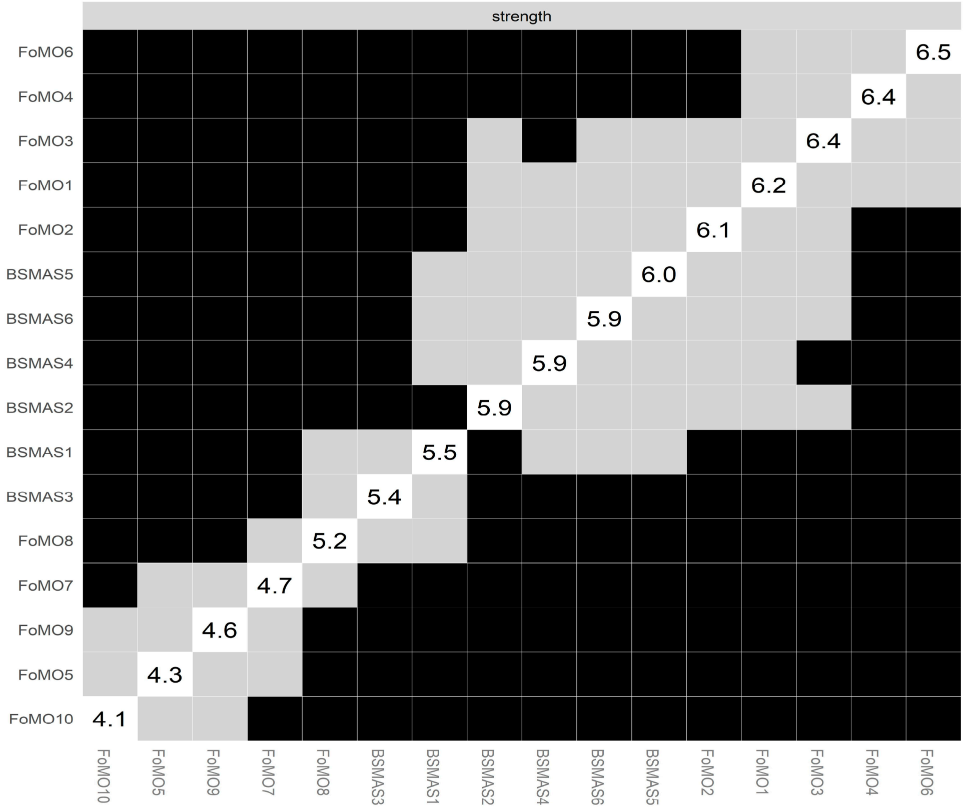

Figure S30. Node strength centrality difference test for combined network in T1.

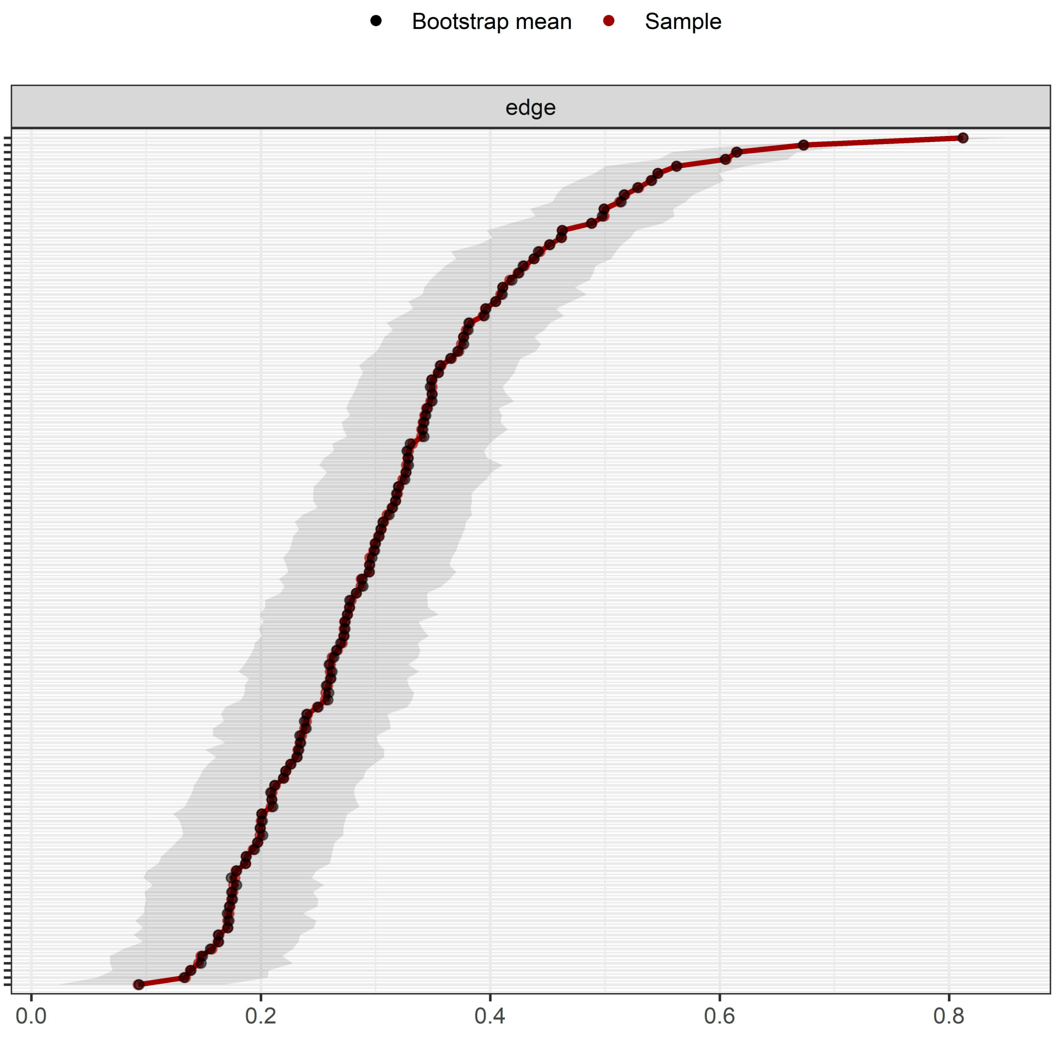

Figure S31. Edge-weight accuracy for combined network in T2.

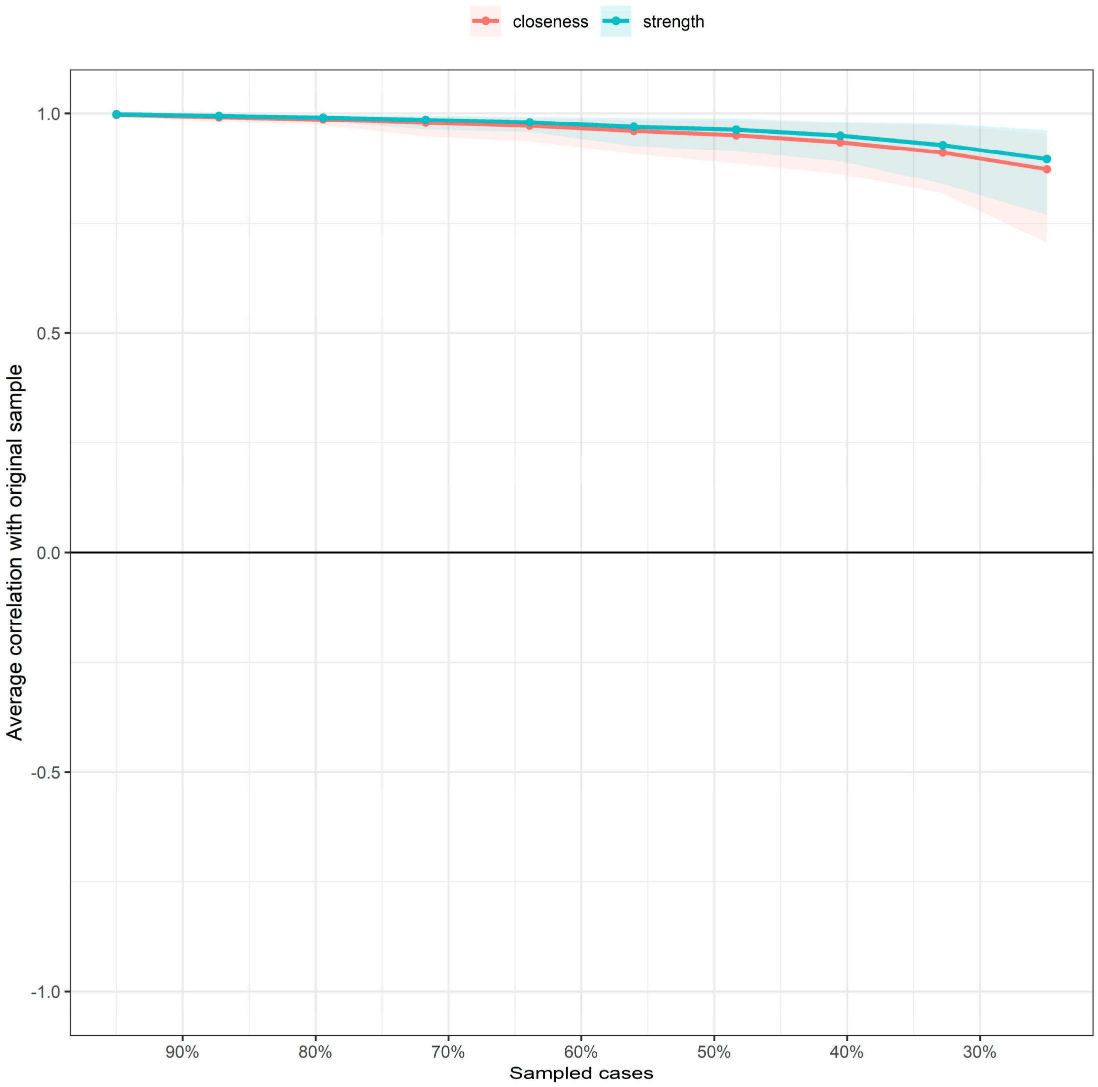

Figure S32. Centrality stability for combined network in T2.

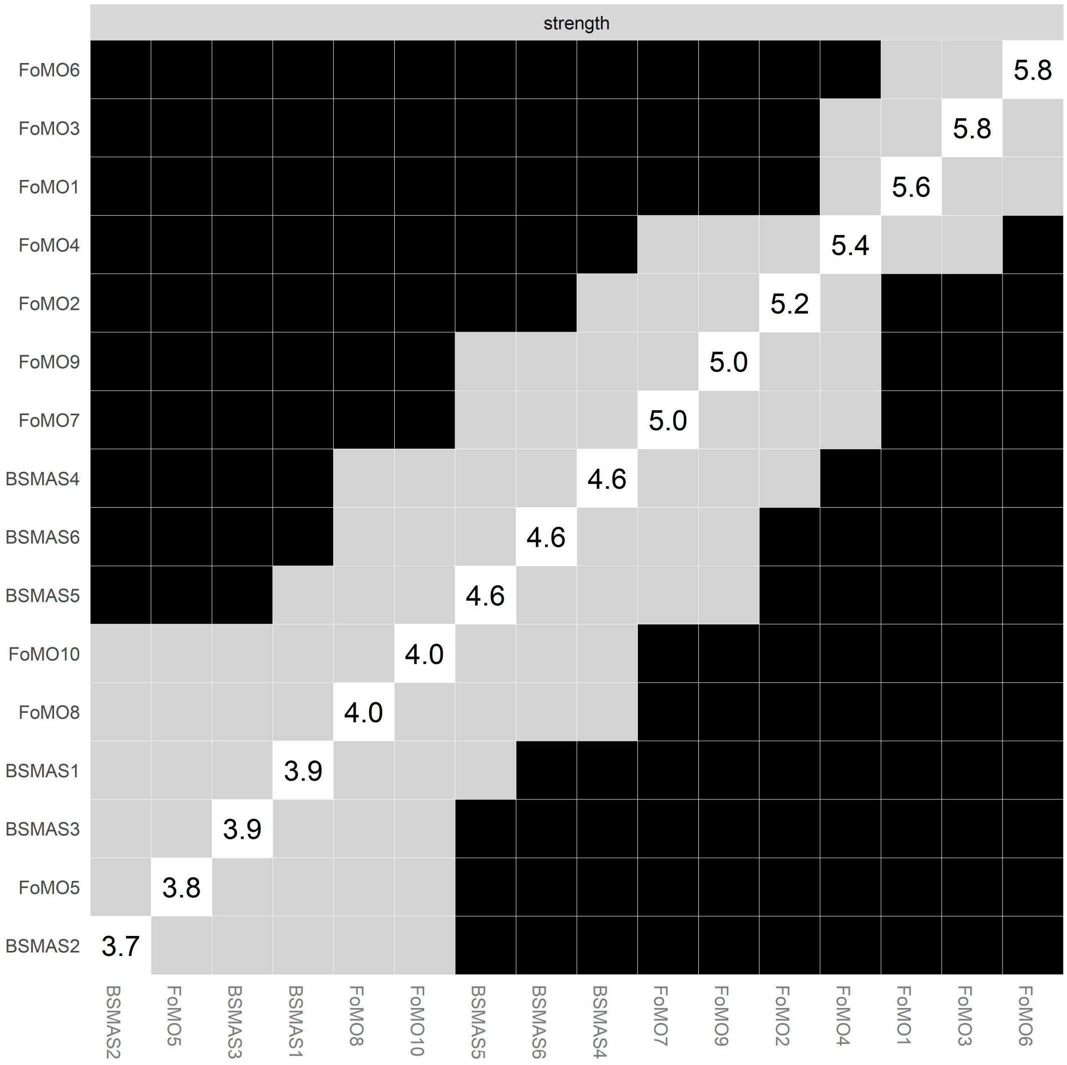

Figure S33. Node strength centrality difference test for combined network in T2.
